# A new HSI denoising method via interpolated block matching 3D and guided filter

**DOI:** 10.7717/peerj.11642

**Published:** 2021-07-27

**Authors:** Ping Xu, Bingqiang Chen, Jingcheng Zhang, Lingyun Xue, Lei Zhu

**Affiliations:** College of Life Information Science & Instrument Engineering, Hangzhou Dianzi University, Hangzhou, China

**Keywords:** IBM3DGF, BM3D, Spectral characteristics, HSI

## Abstract

A new hyperspectral images (HSIs) denoising method via Interpolated Block-Matching and 3D filtering and Guided Filtering (IBM3DGF) denoising method is proposed. First, inter-spectral correlation analysis is used to obtain inter-spectral correlation coefficients and divide the HSIs into several adjacent groups. Second, high-resolution HSIs are produced by using adjacent three images to interpolate. Third, Block-Matching and 3D filtering (BM3D) is conducted to reduce the noise level of each group; Fourth, the guided image filtering is utilized to denoise HSI of each group. Finally, the inverse interpolation is applied to retrieve HSI. Experimental results of synthetic and real HSIs showed that, comparing with other state-of-the-art denoising methods, the proposed IBM3DGF method shows superior performance according to spatial and spectral domain noise assessment. Therefore, the proposed method has a potential to effectively remove the spatial/spectral noise for HSIs.

## Introduction

In the process of acquiring HSIs, spectral characteristics are prone to be distorted under the affection of noise. If an absorption feature is detected, the noise level is expected to be significantly lower than the feature in magnitude (Zhang, 2016). Noise not only reduces the visual quality, but also limits the interpretation of the image, the accuracy of analysis and extraction of spectral information etc.

There exists denoising methods in the spectral domain. Bourguignon, Mary & Slezak (2010) proposed a restoration procedure that operates on each spectrum by minimizing a penalized data-fit criterion. By taking the noise spectral distribution into account, additional constraints can express prior sparsity information in a union of bases. Spectral smoothing filter for removing noise from the hyperspectral data was proposed (Vaiphasa, 2006). Yang, Wang & Zhao (2015) proposed a fluorescence spectrum denoising method for low concentration petroleum pollutants combining the empirical model decomposition (EMD) and the lifting wavelet transform (LWT). Chen, Lu & Peng (2012) proposed a normalized least mean square (NLMS) adaptive filtering method to deduct the noise from the near-infrared spectrum. Liu et al. (2012) and Li et al. (2010) used a combination of wavelet package transformation and other filters for infrared spectrum. Axell & Larsson (2009) proposed a Bayesian approach for spectrum sensing denoising. Lin, Wang & Yao (2011) applied wavelet-based denoising for near infrared spectral spectrum. Yang et al. (2014) proposed a wavelet-based threshold (WT) denoising method for the self-developed Pushbroom Imaging Spectrometer. However, it should be noted that most existed spectral denoising methods ignore the spatial correlation and destroy the image details (Bourguignon, Mary & Slezak, 2010; Vaiphasa, 2006; Yang, Wang & Zhao, 2015; Chen, Lu & Peng, 2012; Liu et al., 2012; Li et al., 2010; Axell & Larsson, 2009; Lin, Wang & Yao, 2011; Yang et al., 2014).

The single-band image denosing methods are another important class of denoisers. Zhang et al. (2008) solved the spatial distribution of the glint contamination according to the fact that the water-leaving radiance value in near-infrared bands was negligible. The method only considered the spatial information, whereas ignored the spectral information. Chen & Qian (2009) proposed a denoising method for HSI using wavelet packets and neighbor wavelet shrinking. Stephan et al. (2008) proposed hyperspectral denoising method by principal component analysis (PCA). To identify cabbages and weeds by the SAM classification, Zu et al. (2015) utilized the minimum noise fraction(MNF) to implement the noise reduction and decorrelation on hyperspectral data. Buades proposed the non-local means (NLM) denoising method which was based on a non local averaging of all pixels in the image (Buades, Coll & Morel, 2005). Dabov et al. (2007) proposed the Block-Matching and 3D filtering (BM3D) denoising method which grouped similar 2-D image fragments into 3-D data and then utilized collaborative filtering to deal with these 3-D groups. The shortcomings of these HSI denoising methods are that the high level of cross-correlation in spectral and spatial dimensions are not well addressed, which lead to the unsatisfactory performance.

The spatial-spectral denoising methods were developed to solve these problems. For the consistent noise intensity of each slice, Atkinson, Kamalabadi & Jones (2003) proposed a Fourier transform denoising method in the spectral domain and a 2D wavelet transform denoising method in spatial domain. Chen, Tien & Adam (2011) proposed a method that denoised all slice of HSIs and processed the 1D spectral signal for each pixel in the spatial domain. A denoising method based on principal component analysis and dictionary learning is proposed to adopt sparse representation and dual-tree complex wavelet transformation to remove noise in the spatial and spectral domains (Huo & Feng, 2014). This method is suitable for the coincident standard deviation of noise in the HSIs, whereas is limited to apply in the situation that the noise standard deviation varies largely among the slices. For the inconsistent noise intensity of each slice, Wang, Xia & Ren (2016) proposed an adaptive denoising method based on the principal component analysis (PCA), noise estimation and dictionary learning. A spatial-spectral hybrid wavelet denoising algorithm was put forward to take advantage of the principle of enhancing noise signal in the spectral differential domain of HSI, which is prone to ignore the detailed characteristics of the spectral curve (Othman & Qian, 2006). In order to maintain the detail characteristics of the hyperspectral remote sensing image, Xu, Sun & Luo (2015) first used noise adjusted principal component analysis (NAPCA) to extract feature and then utilized wavelet transform to denoise low energy component which comes from NAPCA transform. Phillips et al. (2009) proposed an unsupervised spatial-spectral hyperspectral imagery denoising approach based on Monte Carlo sampling (MCS) technique. Their approach allows the incorporation of both spatial and spectral information for hyperspectral imagery denoising. Moreover, it addresses the noise variance heterogeneity effect among different hyperspectral imagery bands. Yuan, Zhang & Shen (2012) proposed a HSI denoising algorithm using a spectral-spatial adaptive total variation (TV) model, in which both the spectral noise differences and spatial information differences are considered in the noise reduction process. In our previous research (Xu et al., 2017b), we proposed a grouped 3D discrete cosine transform (G3DDCT) to solve the problems that noise intensity of each band for plant hyperspectral image is different and noise exists in both spatial and spectral domains.

Recently, Karami, Heylen & Scheunders (2015) proposed an effective method to distinguish between bands with low levels of Gaussian noise (LGN bands) and bands with mixed noise (MN bands) based on a spectral correlation procedure. Yang et al. (2016) proposed a sparse representation framework that unifies denoising and spectral u nmixing in a closed-loop manner. Chen & Qian (2011) proposed a method to decorrelate the image information of HSI cubes by using principal component analysis (PCA) and removing the noise in the low-energy PCA output channels. Chen et al. (2017) present a nonconvex low-rank matrix approximation (NonLRMA) model and the corresponding HSI denoising method by reformulating the approximation problem using nonconvex regularizer. Comparing with the traditional nuclear norm, their results found a better approximation of the original sparsity regularised rank function. Liu, Bourennane & Fossati (2012a) exploit the parallel factor analysis (PARAFAC) denoising method. An HSI denoising method is proposed by jointly utilizing the global and local redundancy and correlation(RAC) in spatial/spectral domains (Zhao & Yang, 2015). Qiao proposed a novel algorithm for HSI feature extraction by exploiting the curvelet-transformed domain via spectral feature processing technique-singular spectrum analysis (SSA) (Qiao et al., 2017). Yuan, Zhang & Shen (2014) proposed a HSI denoising algorithm with a spatial-spectral view fusion strategy. A novel spectral-spatial adaptive sparse representation (SSASR) method is proposed for HSI denoising (Lu et al., 2016). He proposed a guided filtering (GF) algorithm which computed the filtering output by considering the content of a guidance image (He, Sun & Tang, 2013). Gu et al. (2017) proposed the weighted nuclear norm minimization (WNNM) denoising method, which adaptively assigned weights on different singular values. They proposed a hyperspectral image denoising method using using local low-rank matrix recovery and global spatial–spectral total variation (He et al., 2018). Zhang proposed a hyperspectral image restoration method using low-rank matrix recovery (LRMR) (Zhang et al., 2014). Sun proposed new endmember extraction methods using parameter subspace clustering constraint and band selection method based on PCA (Sun et al., 2017a; Sun et al., 2017b; Sun & Du, 2018). Zhuang & Bioucas-Dias (2018) proposed a hyperspectral image denoising and inpainting method based on low-rank and sparse representations (FastHyde and FastHyin). Most above research topics are on the simulated HSIs. In recent years, there are some denoising studies which put emphasis on realistic HSIs (Gao et al. 2013a; Hao et al., 2016).

However, most of the above denosing methods were seldom to do correlation analysis to get the characteristics of HSIs. As we know, it is nearly impossible for a denosing method to obtain the optimal performance for any kind of HSIs. For specific HSIs, to obtain the most appropriate method should be a better choice according to their characteristics. Therefore, in order to enhance the adaptive denoising performance in spatial/spectral dimensions for different noise intensity in each band of HSIs, a new HSI denoising method of Block-Matching and 3D filtering and Guided Filtering (IBM3DGF) is proposed in this paper. Inter-spectral correlation analysis is first done to divide the HSIs into several adjacent groups. High-resolution HSIs are then produced by using adjacent three images to interpolate and Block-Matching and 3D filtering (BM3D) is conducted to reduce the noise level for each group. Guide filter technology is introduced to enhance the denoising performance of each group.

## The Proposed IBM3DGF Method

**Figure 1:**
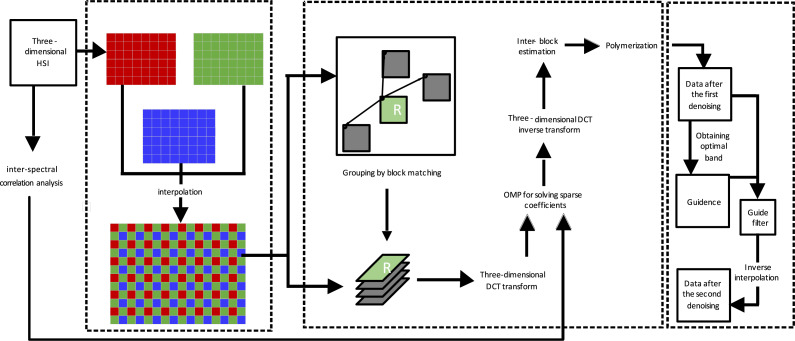
The procedure flow of the IBM3DGF.

IBM3DGF can be divided into the following four parts: inter-spectral correlation analysis, interpolation, modified BM3D and guided filtering. Inter-spectral correlation analysis is performed to divide the whole HSIs into different adjacent groups in which bands show high correlation coefficients. Interpolation is induced to obtain high resolution HSIs by using three adjacent images. Modified BM3D, which only retain its first step and omit its second step of Wiener filtering because of the following guide filtering process has strong denoising performance. The guided filtering is utilized to further remove the noises in each group. First, adjacent three bands were combined into an interpolation image; second, similar blocks were searched by non-local mean denoising (NLM) and stacked into 3D cubic; third, the sparse representation denoising algorithm of 3D DCT dictionary was used to denoise the 3D cubic, and the initial denoised plant HSIs were obtained; fourth, noise estimation was carried out on initial denoised plant HSIs using the local mean standard deviation method, the optimal band was selected as the guide image, and the guide filter was performed to obtain the secondary denoised HSIs; Finally, the final plant denoised HSIs is subsampled from the secondary denoised HSIs according to the inverse interpolation process. Figure 1 shows the procedure of IBM3DGF method as follows:

(a) inter-spectral correlation analysis: the whole HSIs can be separated into several adjacent groups in which bands show high correlation coefficients according to inter-spectral correlation analysis;

(b) interpolation: high resolution HSIs can be obtain by interpolation using adjacent three bands;

(c) modified BM3D

(c.1) inter-block estimation:

(i) A block matching is used to search the optimal similar blocks and then be combined into the 3D array to enhance their sparsity;

(ii) 3D DCT transform is carried out;

(iii) Orthogonal Matching Pursuit(OMP) algorithm is used to calculate the coefficients iteratively in which adaptive threshold in (a) is regarded as the hard threshold;

(iv) inversed 3D DCT transform is done to realize inter-block estimation;

(c.2) polymerization: the denoised images can be obtained by weighted average calculation;

(d) guided filtering

(d.1) using local mean standard deviation to estimate the noise, and choose the optimal band in each group which has highest PSNR and reserve good border edge as the guided image;

(d.2) utilizing the guided image filtering to denoise hyperspectral images per band for each group for the second time;

(e) inverse interpolation to recover HSIs;

In order to improve the sparsity and transform the search process of 3D similar block into that of 2D array, adjacent three bands are combined into an interpolation image. Figure 2 shows the interpolation process, in which red image is the former band, green image is the current band, and blue image is the next band. In the interpolation image, current band holds the double amount of information of the former and next image. The resolution of the interpolation image improved by four times, and its sparsity can also be improved. Figure 3 shows the reverse interpolation process, which transforms the high-resolution image into low resolution one:

(1) \begin{eqnarray*}{G}^{{^{\prime\prime}}}i=({G}^{{^{\prime}}}i+{G}^{{^{\prime\prime}}}i)/2\end{eqnarray*}

**Figure 2:**
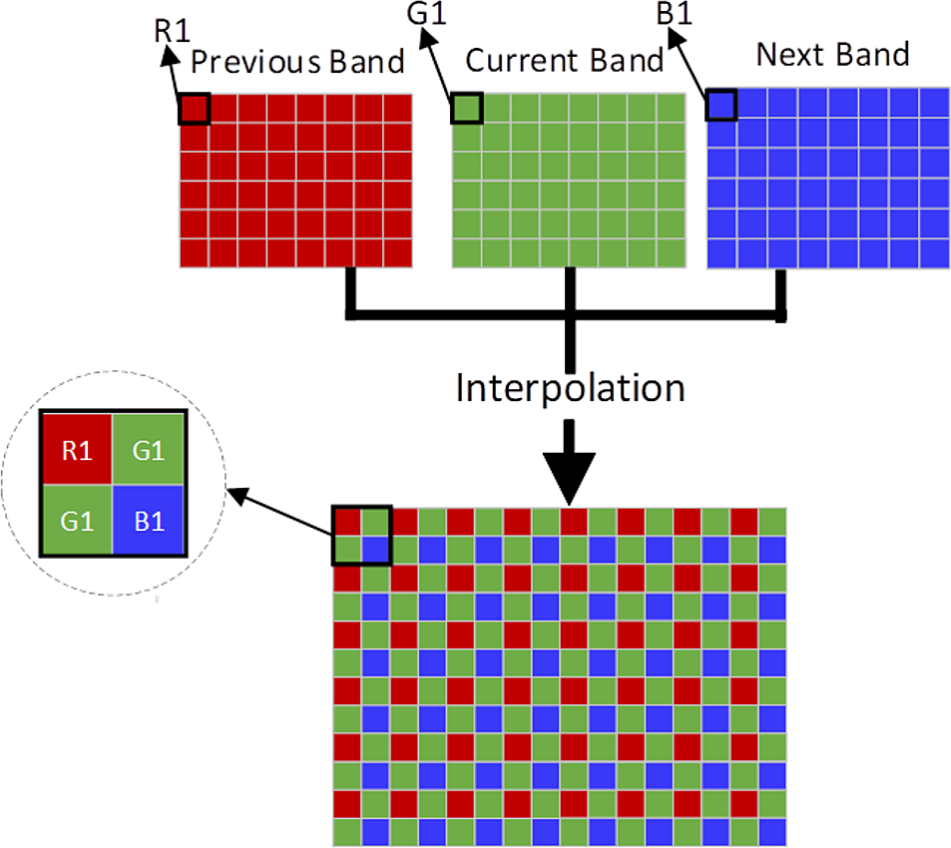
The interpolation of HSI.

**Figure 3:**
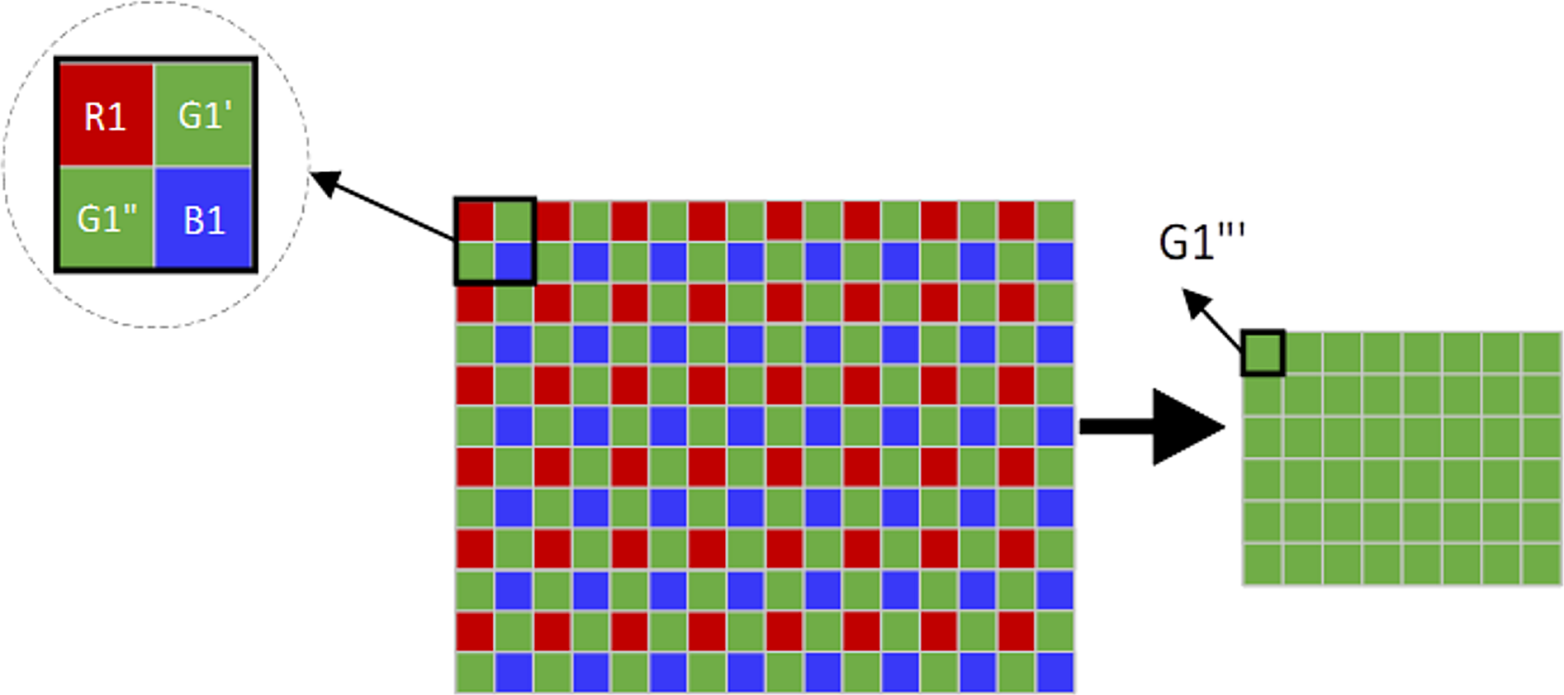
The reverse interpolation of HSI.

where *i* is the *i* th pixel.

Guided image filtering was proposed by He in 2010 (He, Sun & Tang, 2013), which can not only preserve the image edge and enhance image details, but also reduce the noise of the image. The guided image needs to obey a linear relationship with the original image.

## Experimental Results

To validate the proposed method, we perform experiments on both the synthetic HSI and real HSI. Comparisons are made between our method and some state-of-the-art methods, including NLM, BM3D, G3DDCT, GF, WNNM, and FastHyde. For NLM method, the searching window of each band is 7 × 7, and the adjacent window is 3 × 3; for BM3D method, the noise standard deviation of each band is chosen as the threshold and the size of match block is 8 × 8; for G3DDCT method, the standard deviation of each band is estimated as the reference threshold and the size of match block is 8 × 8. For IBM3DGF method, the size of a match block is 8 × 8 and the local window is 3 × 3.

### Synthetic HSI experiments

#### Indian pines

The first synthetic HSIs are generated according to the ground truth of Indian Pines data (Zhang, Du & Zhang, 2015) (Qian & Ye, 2013). The 17 endmembers are chosen from the USGS digital spectral library ‘splib06a’ (Camps-Valls et al., 2006). Sixteen classes of labeled pixels are replaced by the signatures from similar endmembers, respectively. And all unlabeled pixels were replaced by the 17th endmember. The size of the image is 145 ×145. The pixel gray level is an 8-bit unsigned integer ranging from 0 to 255. The wavebands over 370 nm–2488 nm are selected, which resulted in a total of 224 slices for the experimental data. This synthetic HSI can be considered as a cleaned data.

##### Spectral characteristics analysis.

**Figure 4:**
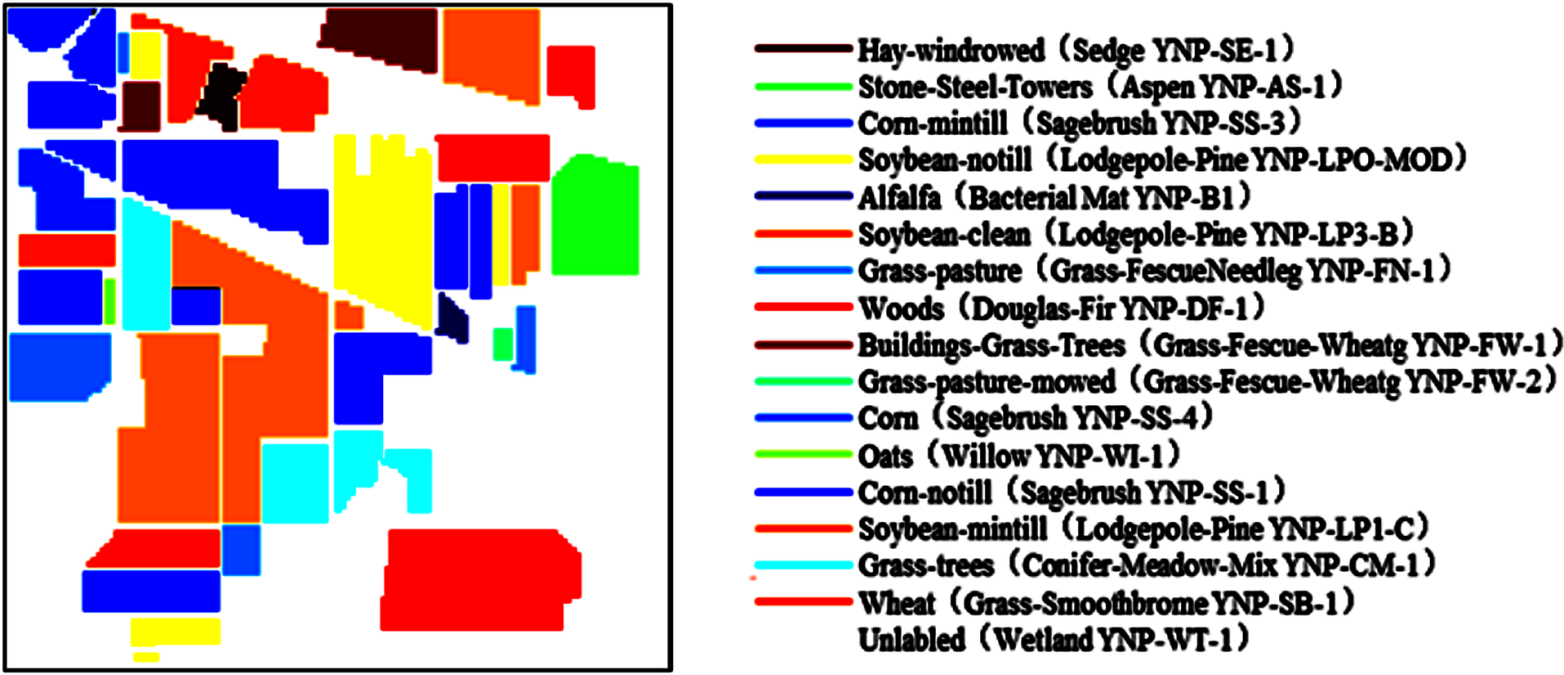
Spatial image of synthetic HSI and spectral labeling for each region.

**Figure 5:**
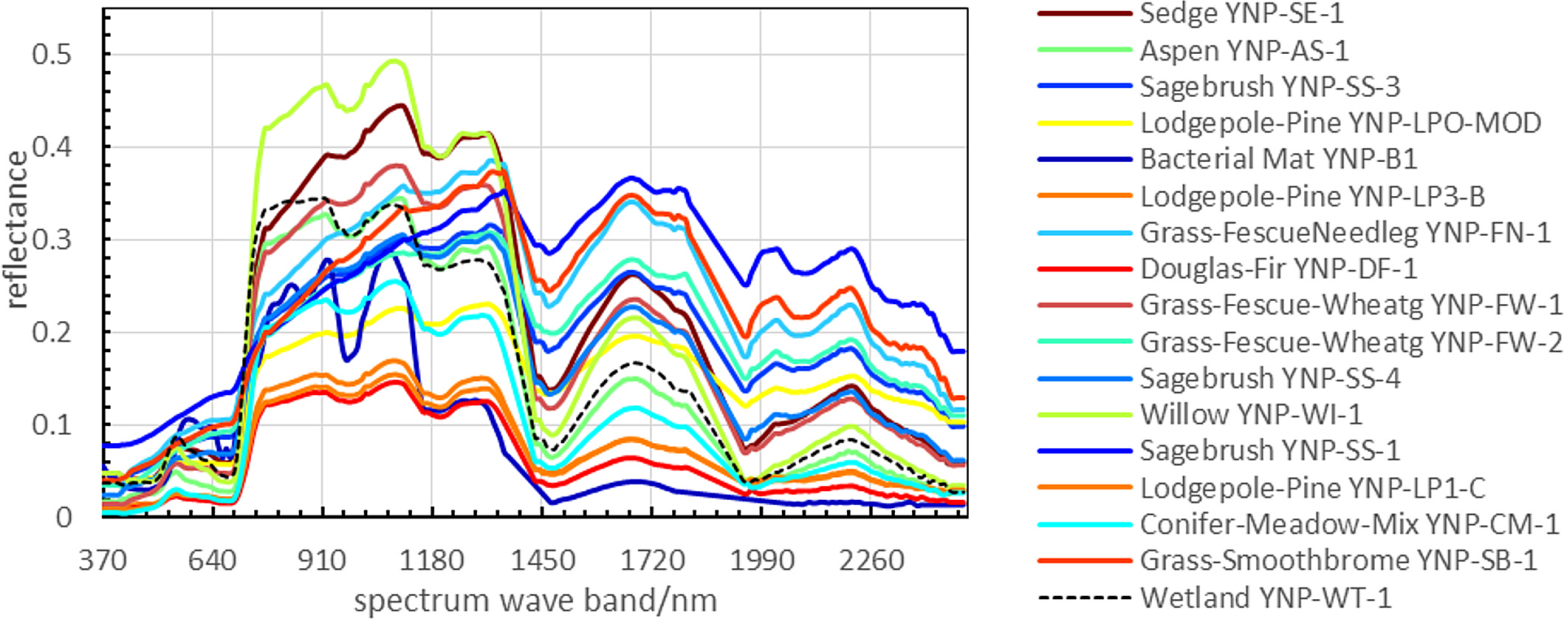
The spectral curves of 17 categories of objects.

The Indian Pines data set includes 16 categories of ground objects and background. In this study, noiseless HSI is shown in Fig. 4. The spectral curves of each region are extracted from the synthetic data as shown in Fig. 5.

**Figure 6:**
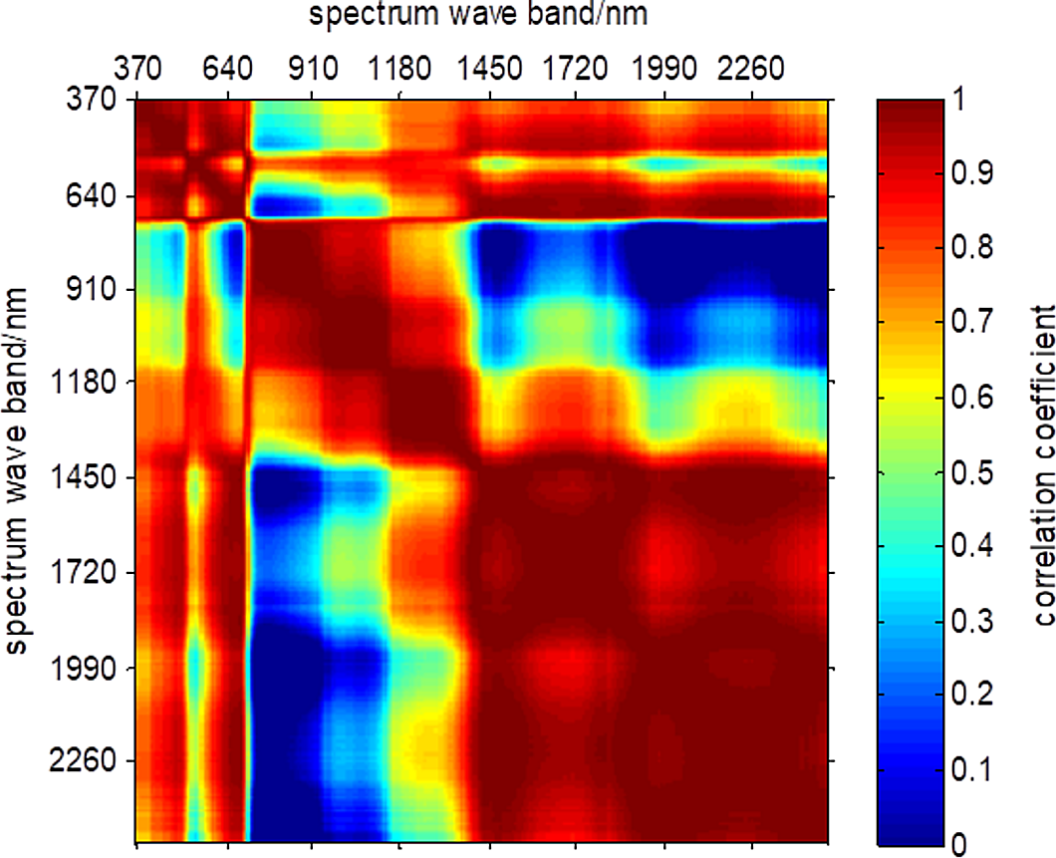
Spectral correlation coefficients of synthetic HSI.

Analysis is conducted on spectral correlation at the waveband range of 370 nm–2,488 nm of HSIs. The result is shown in Fig. 6. The correlation of spectral curve is relatively high over the range of 370 nm–670 nm, synthetic 90 nm–1,310 nm and 1,970 nm–2,488 nm, and relatively low over the range of 670 nm–790 nm and 1,310 nm–1,970 nm. Besides, the correlation among these five ranges is relatively low.

##### The additive noise analysis.

**Figure 7:**
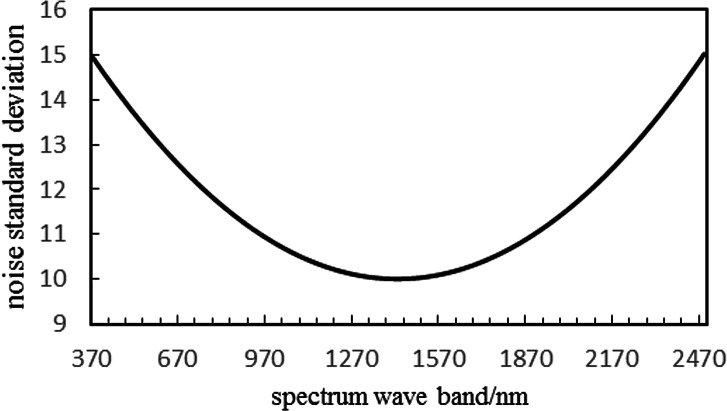
Noise standard deviation curve of concave parabolic trend.

Assuming the noise is zero-mean additive Gauss white noise, for the synthetic data, the following two types of noise are applied in this paper: (1) Noise with constant standard deviation. All bands have the same noise intensity and the noise standard deviation *σ* is 15. (2) Noise with nonconstant standard deviation. The noise intensity of each band shows a concave parabolic tendency, with average noise standard deviation $\overline{\sigma }$of 11.6, as shown in Fig. 7.

##### Evaluation of denoising performance in the spatial domain.

**Figure 8:**
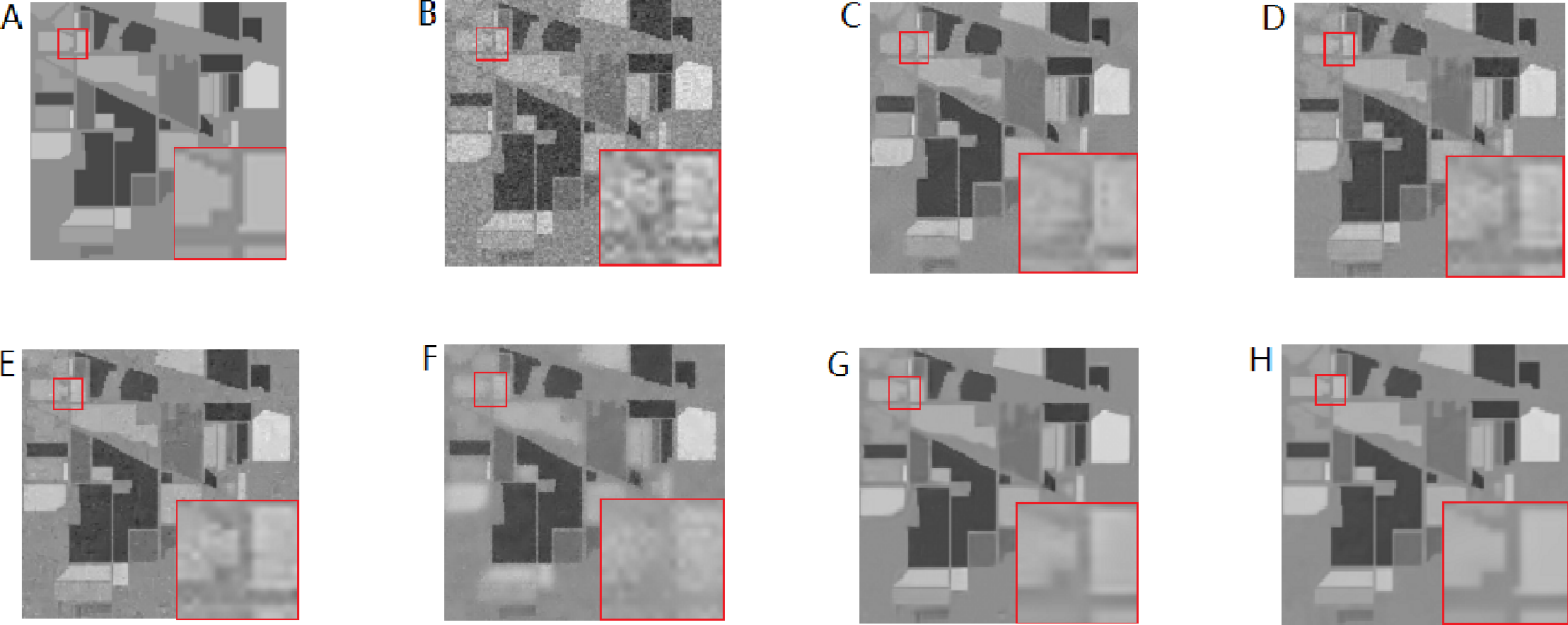
Comparison of denoising results of the noise of same variance for different methods of 950 nm band.

The above methods are used to denoise the synthetic HSI after adding the noise with the same variance. Here the images of 950 nm are used to make comparisons. From Figs. 8A–8G, In turn, no noise image, noisy image, NLM denoising image, BM3D denoising image, G3DDCT denoising image, GF denoising image, WNNM denoising image, IBM3DGF denoising image), it can be seen that the proposed IBM3DGF method yields better denoising results, comparing with the other methods. In the IBM3DGF denoising result, not only the noise is thoroughly suppressed, but also the edge information and the detailed information are well preserved. In the result of NLM denoising method, some “artifacts” are produced with the image, and the edge information is also not well preserved. BM3D is one of the best denoising methods in designing for single image. G3DDCT divides the HSIs into groups and enhances the sparsity of HSIs. IBM3DGF induces the guided image filtering and combines the advantages of BM3D and G3DDCT denoising methods. IBM3DGF and WN nm can achieve better denoising performance than the other methods. From comparisons of the recovering partial enlargement for different denoising methods, IBM3DGF can preserve the details and edge information of image better than the other denoising methods. Therefore, comparison with the other denoising methods, the IBM3DGF method can not only maintain better edge information, but also suppress the noise information more effectively.

**Figure 9:**
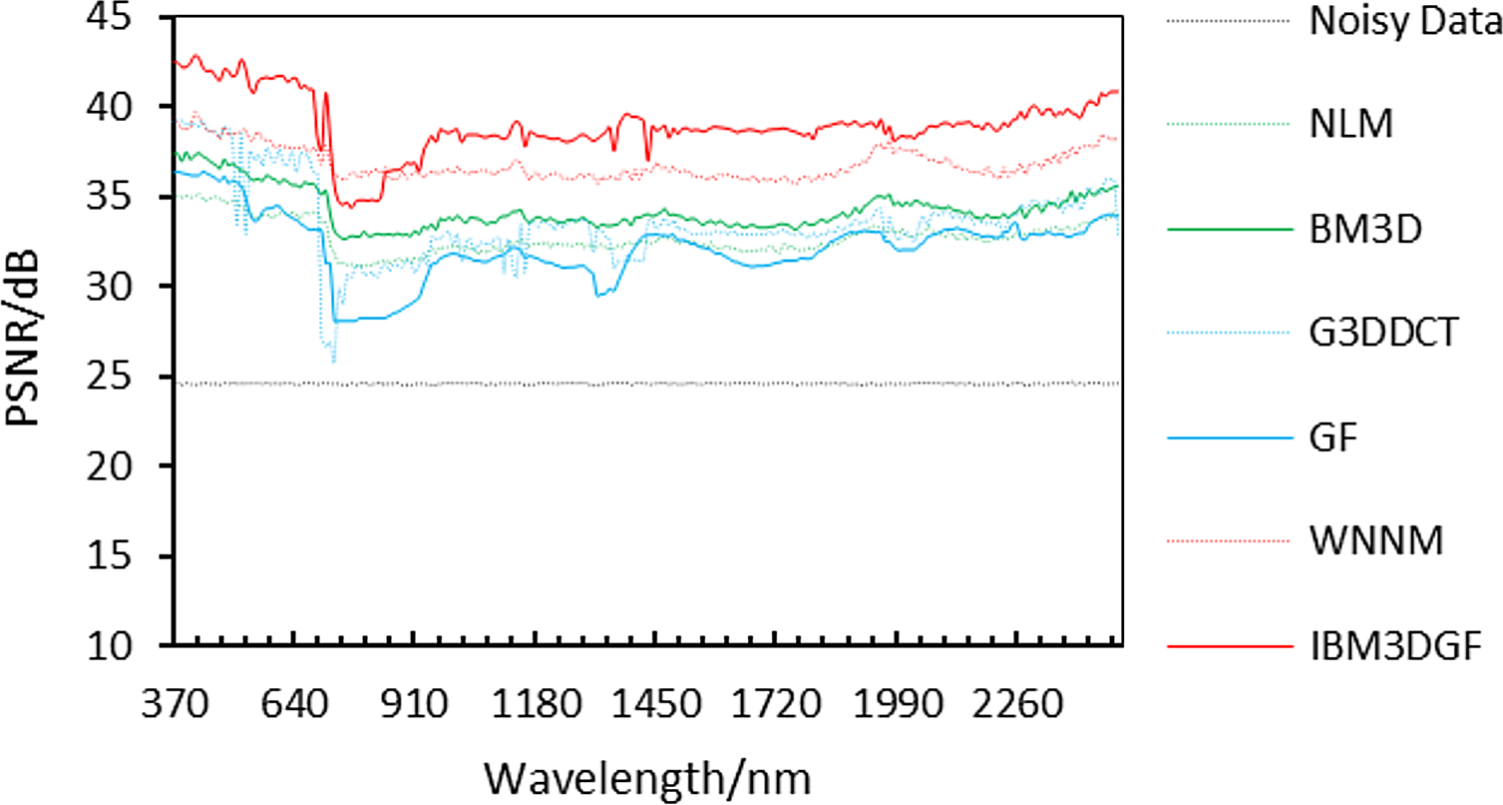
Curves of PSNR of the noise of the samevariance for different methods.

The denoising evaluation of adding noise has the same variance to the synthetic HSI. The detailed per-band performance of the proposed method is plotted in Fig. 9, together with the performance of the other methods. The proposed IBM3DGF method shows PSNR-per-band that is higher than the other methods for almost all 224 bands in 370 nm–2,488 nm.

**Figure 10:**
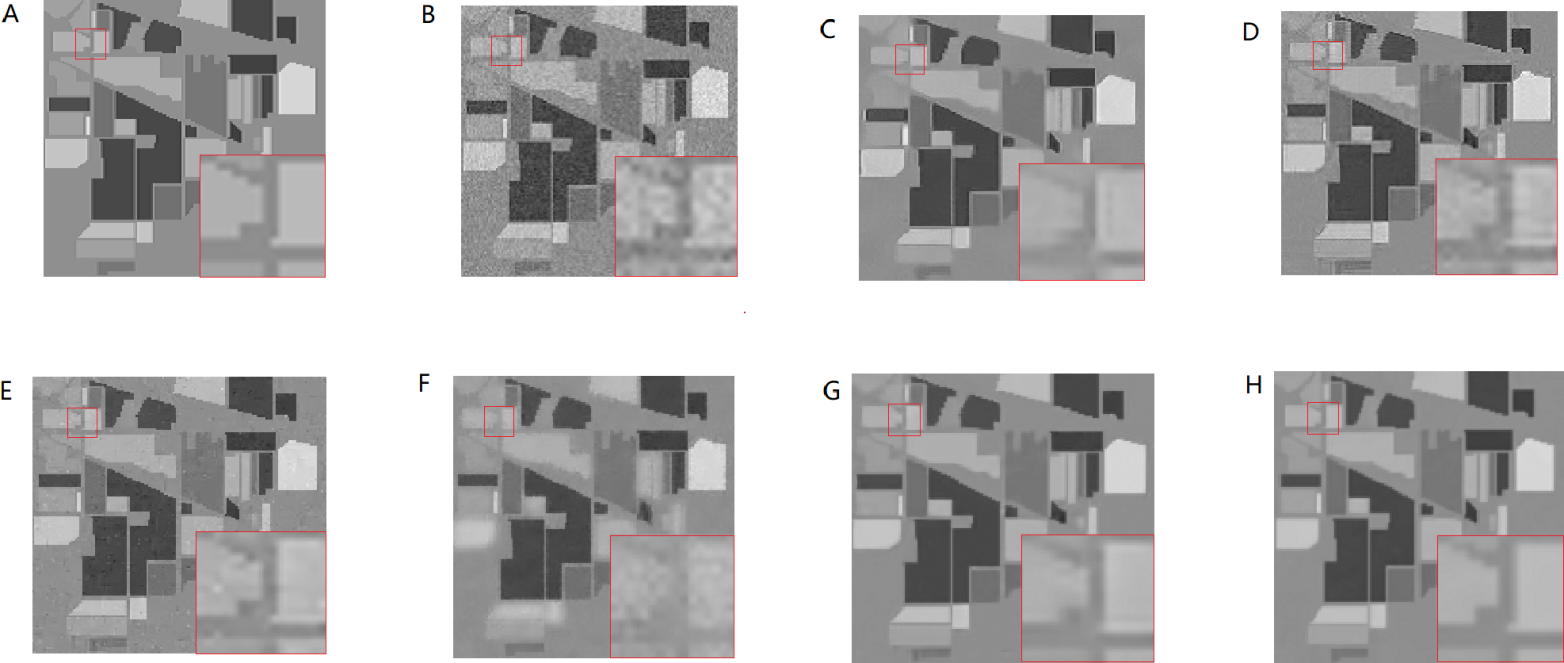
Comparison of denoising results of the noise of different variance for different methods of 950 nm band.

**Figure 11:**
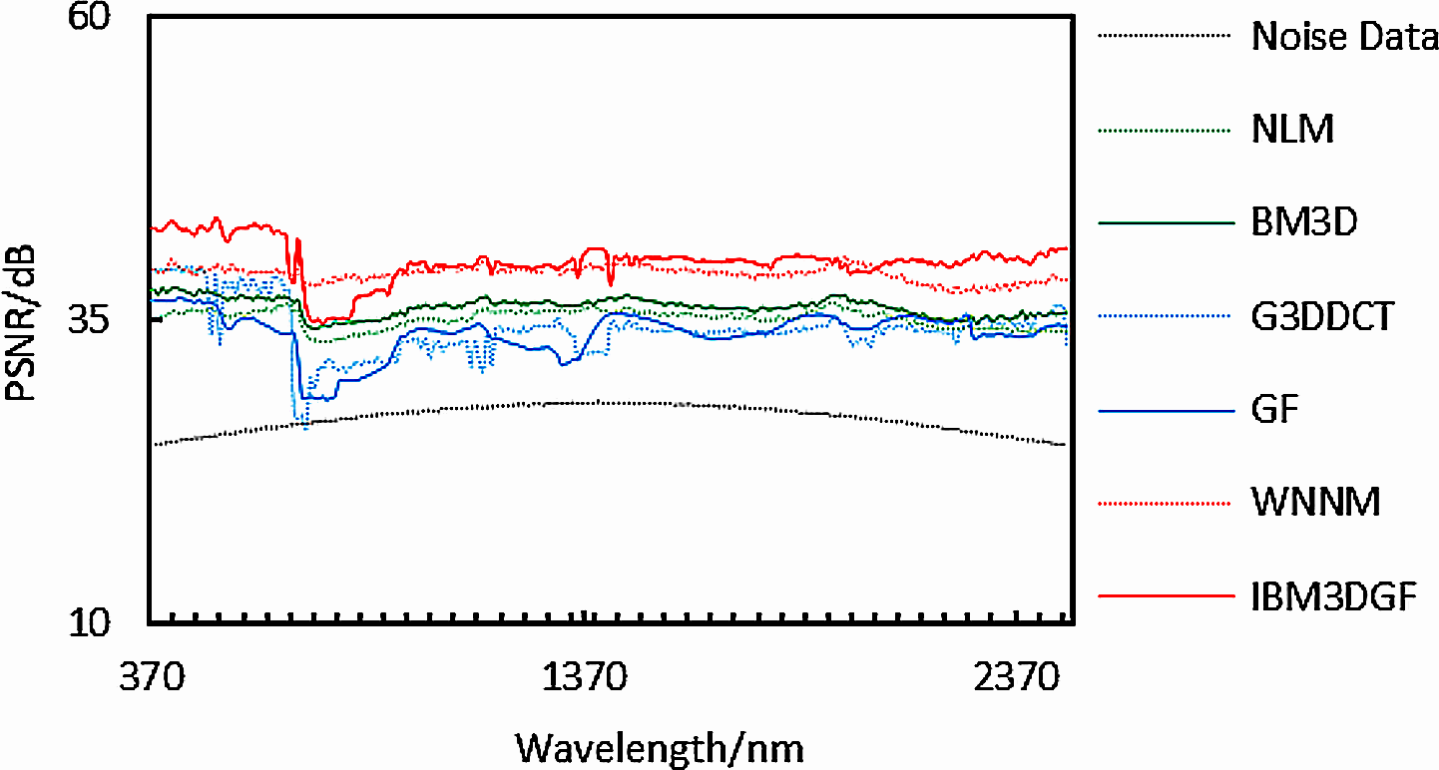
Curves of PSNR of the noise of differentvariance for different methods.

The above methods are used to denoise the synthetic HSI after adding noise with the different variance. Here the images of 950 nm are used as examples to make a comparison. From Figs. 10A–10H), it can be seen that the proposed IBM3DGF method produces better denoising results for noise under different variance than other methods. Similar to the experimental results of the noise of same variance, in the comparisons of the recovering partial enlargement for different denoising methods, IBM3DGF can also preserve the details and edge information of image better than the other denoising methods. The detailed per-band performance of the proposed method is plotted in Fig. 11, together with the performance of the other methods. The proposed IBM3DGF method shows PSNR-per-band that is also significantly higher than the other methods for most bands in 370–2488 nm. As it can be seen that the proposed IBM3DGF method has good adaptive denoising performance for noise under different variance.

The comparison of the average PSNR in the spatial domain for noise with the same variance and different variance are shown in Table 1. The average PSNR of the IBM3DGF is significantly higher than the other methods. For noise of the same variance, the average PSNR of IBM3DGF is 4.7dB and 2.14dB higher than that of BM3D and WNNM, respectively. For noise of different variance, the average PSNR of IBM3DGF is 3.77dB and 1.03dB higher than that of BM3D and WN nm, respectively. The proposed IBM3DGF method mainly includes two parts of Interpolated Block Matching 3D(IBM3D) and GF. As it can be seen, for the hyperspectral images with noise of same variance and different variance, the denoising performance of IBM3D can achieve 5.49dB and 4.24dB higher values of PNSR than those of GF, respectively. Therefore, IBM3D plays more effective phase than that of GF in the spatial domian.

**Table 1:** Comparison of the PSNR in the spatial domain.

Different methods	PSNR for the noise of same variance(dB)	PSNR for the noise of different variance(dB)
Noisy data	24.06	26.86
NLM	32.75	35.09
BM3D	34.27	36.05
IBM3D	37.72	38.13
G3DDCT	33.75	34.34
GF	32.23	33.89
WNNM	36.83	38.79
IBM3DGF	38.97	39.82

The rice noise is also involved to evaluate the denoising performance. Standard deviation *σ* varies from 5% to 30%. Table 2 shows the mean PSNR comparison of BM3D, WNNM, and IBM3DGF for different values of standard deviation *σ* of rice noise in the spatial domain. The IBM3DGF can achieve higher values of mean PSNR than those of BM3D and WN nm at different values of standard deviation *σ*.

**Table 2:** Comparison of the PSNR for rice noise in the spatial domain/dB.

Different methods	*σ* = 5%	*σ* = 10%	*σ* = 15%	*σ* = 20%	*σ* = 25%	*σ* = 30%
BM3D	43.14	37.09	33.12	30.05	27.58	25.47
WNNM	44.61	37.94	33.63	30.40	27.62	25.48
IBM3DGF	46.06	39.57	35.08	31.53	28.73	26.38

##### Evaluation of denoising performance in the spectral domain.

**Figure 12:**
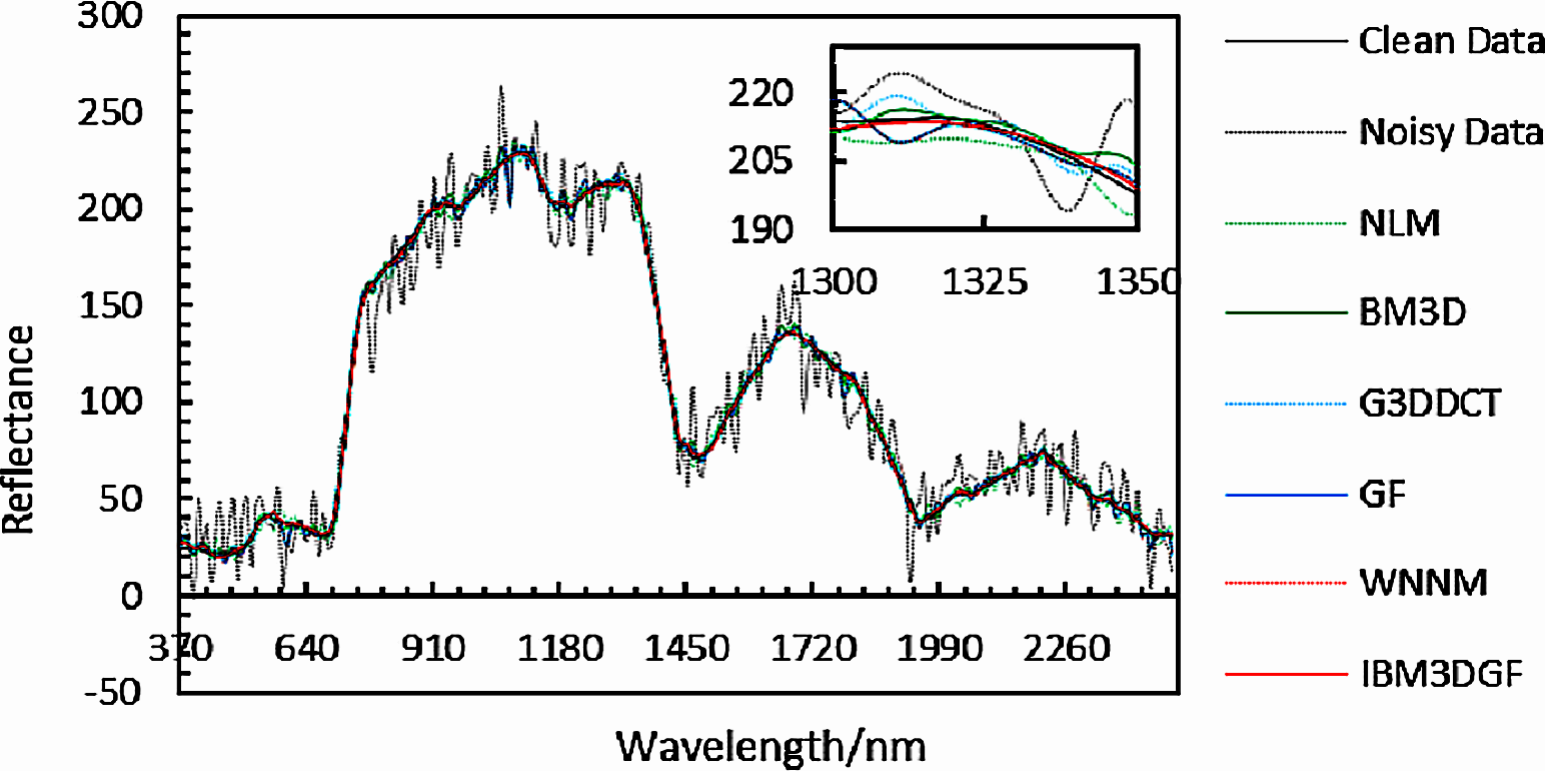
Comparison of spectral curves at *f* (50,130, *z*) for adding noise of the same variance.

**Figure 13:**
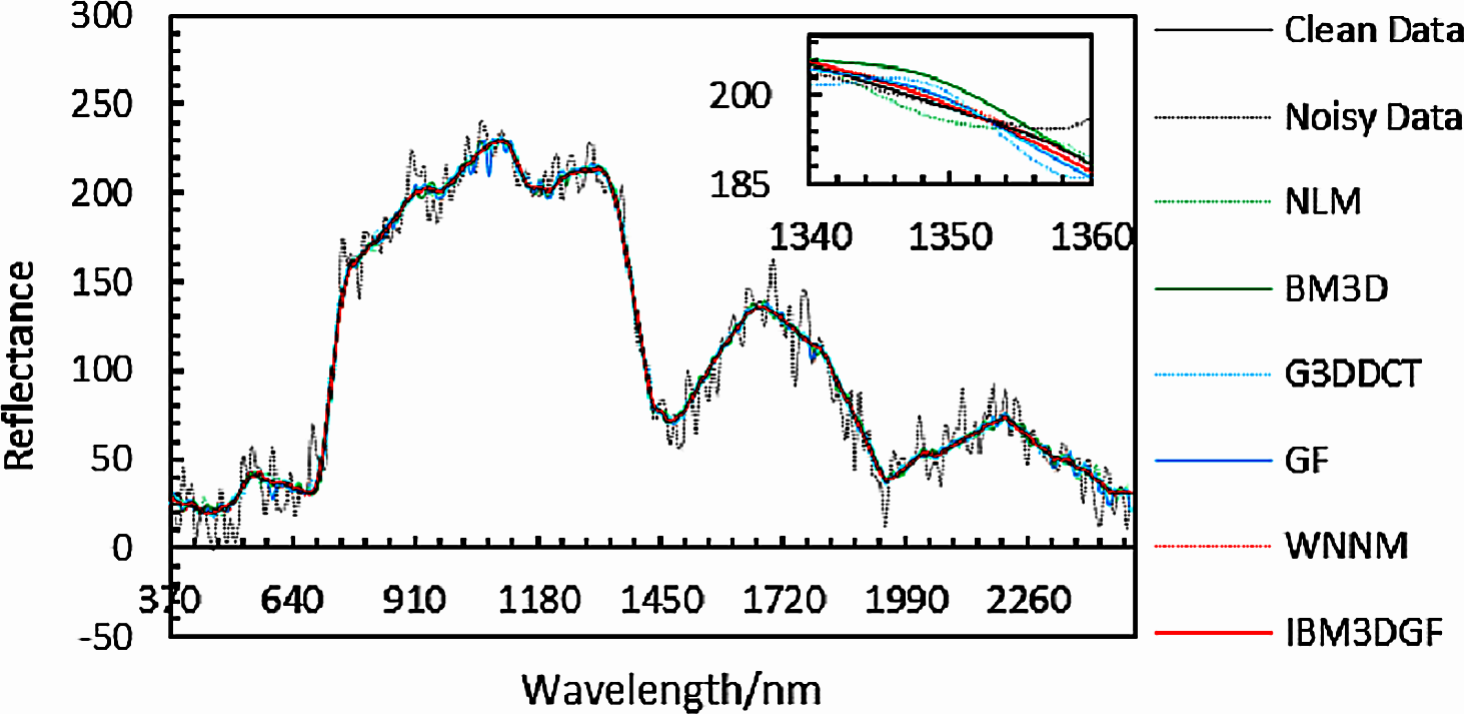
Comparison of spectral curves at *f* (50,130,*z*) for adding noise of different variance.

The experimental results of denoised spectral curves at *f*(50, 130, *z*) under noise with the same variance and different variance for different methods are provided in Figs. 12 and 13. Local images of spectral curves in the 370 nm–2,488 nm are magnified to be shown in the upper right corner of these figures. It is shown that the spectral curves of IBM3DGF for noise with the same variance and different variance are all closer to the original curves than those of the other methods. IBM3DGF not only takes the advantage of the high inter-spectral correlation and inter-spatial correlation, but also introduces a guided image filter to refine the details of the HSIs.

The comparison of the root mean square error (RMSE) in the spectral domain for noise with the same variance and different variance is shown in Table 3. For noise with same variance, the RMSE of the IBM3DGF is 0.86 lower than that of WNNM. For noise with different variance, the RMSE of IBM3DGF is 0.68 lower than that of WNNM. Therefore, the spectral denoising results show that IBM3DGF achieves the best performance for noise with the same variance and different variance. Besides, for the hyperspectral images with noise of same variance and different variance, the denoising performance of IBM3D can achieve 4.15 and 3.77 lower values of RMSE than those of GF, respectively. Therefore, IBM3D plays more effective phase than that of GF in the spectral domain.

**Table 3:** Comparison of the RMSE in the spectral domain.

Different methods	RMSE for the noise of same variance(dB)	RMSE for the noise of different variance(dB)
Noisy data	21.70	17.02
NLM	7.44	5.05
BM3D	6.22	5.03
IBM3D	3.68	2.91
G3DDCT	5.70	5.08
GF	7.83	6.68
WNNM	3.77	3.05
IBM3DGF	2.81	2.37

Table 4 shows the RMSE comparison of BM3D, WN nm, and IBM3DGF for different values of standard deviation *σ* of rice noise in the spectral domain. IBM3DGF can achieve lower values of mean RMSE than those of BM3D and WN nm at different values of standard deviation *σ*. Therefore, IBM3DGF can acquire better denoising performance in spectral domain.

**Table 4:** Comparison of the RMSE for rice noise in the spectral domain.

Different methods	*σ* = 5%	*σ* = 10%	*σ* = 15%	*σ* = 20%	*σ* = 25%	*σ* = 30%
BM3D	2.09	4.84	8.13	11.72	15.40	18.92
WNNM	1.87	4.61	7.96	11.56	15.30	18.79
IBM3DGF	1.84	4.25	7.50	11.12	14.83	18.36

#### Pavia University

The second synthetic HSIs are acquired by the ROSIS sensor during a flight campaign over Pavia University in northern Italy. The number of spectral bands is 103 and the size of the image is is 610 ×610 pixels. A part of Pavia University HSIs with the size of 256 × 256 ×103 are extracted for the experiment.

**Figure 14:**
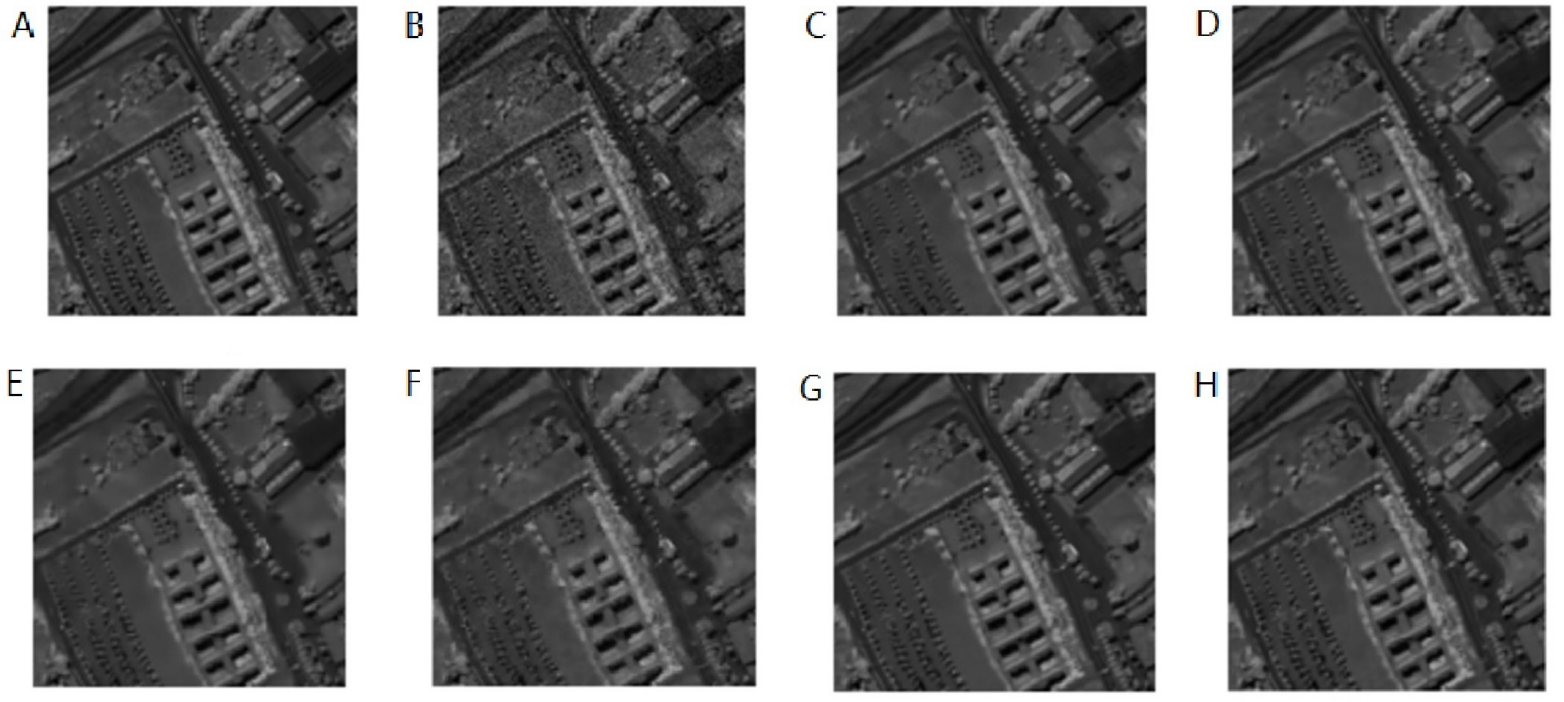
Comparison of denoising results on Pavia University with noise standard deviation of 15.

**Figure 15:**
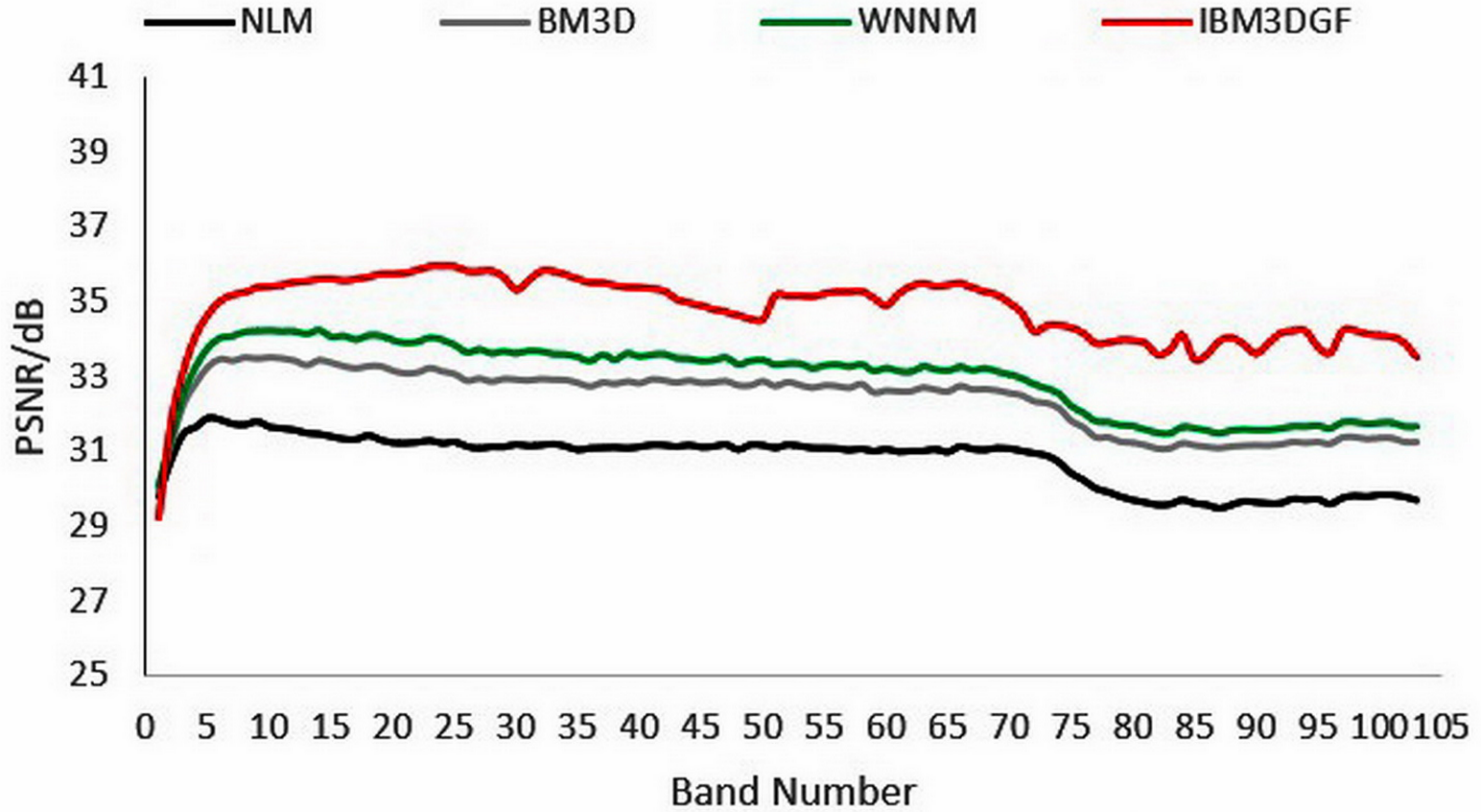
PSNR after denoising of Pavia University with different noise standard deviations.

In the experiment, the noise is zero-mean additive Gauss white noise, and all bands have zero-mean additive Gauss white noise with the same intensity and differernt noise standard deviation *σ* of 6,8,10,15, and 20. Figure 14A–14I), In turn, no noise image, noisy image, NLM denoising image, BM3D denoising image, G3DDCT denoising image, GF denoising image, WNNM denoising image, IBM3DGF denoising image), shows the denoising results of the 6th band of different denoising algorithms for the noise standard deviation *σ* of 15. The detailed per-band PSNR performance of different denoising methods for the noise standard deviation *σ* of 15 are plotted in Fig. 15. Table 5 shows the comparison of the average PSNR in the spatial domain of different algorithms for Gaussian noise with different standard deviations. It can be seen from Figs. 14 and 15 and Table 5 that the proposed IBM3DGF denoising can achieve significantly better denoising performance than that of the other denoising algorithms for zero-mean additive Gauss white noise with different noise standard deviation.

### Real HSI experiments

#### Spectral characteristics analysis

**Figure 16:**
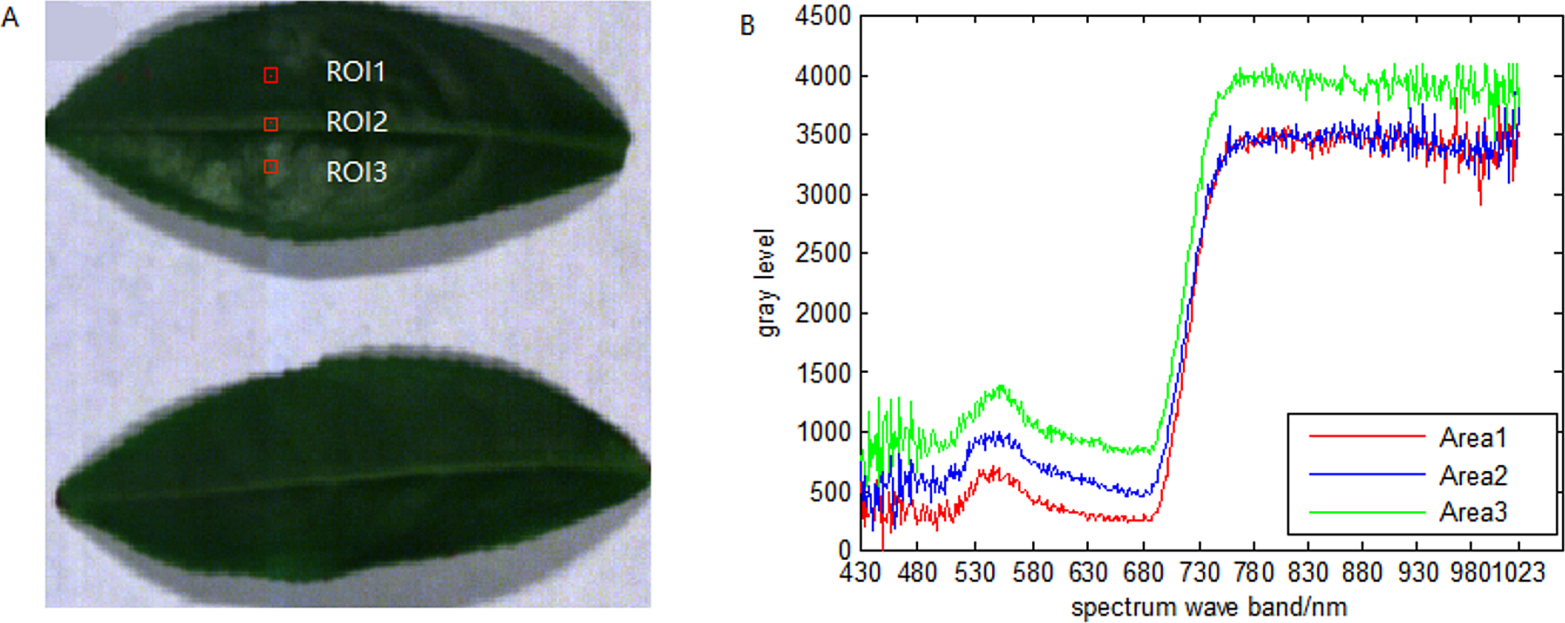
Spatial image of HSI and mean spectral curves of ROIs.

The plant hyperspectral images in our previous research (Xu et al., 2017a) are used in this paper. HSIs of 12 pieces of camellia sinensis of 320 bands in the wavelength of 450 nm–850 nm and are chosen to perform the following experiments. Within these HSIs, a single pixel is defined by a 12-bit unsigned integer. 661 nm, 553 nm and 449 nm are selected from red, green, blue channels, respectively, to form a RGB image as shown in Fig. 16A. 3 ROIs are selected from the original image, and the mean spectral curve of each ROIs was shown in Fig. 16B. It can be seen that the HSIs of 430 nm–530 nm and 930nm–1,023 nm wavebands are seriously affected by noise.

**Table 5:** Comparison of the average PSNR in the spatial domain for Gaussian noise with different standard deviations.

Methods	PSNR/dB				
	Noise standard deviation				
	6	8	10	15	20
NLM	32.90	32.76	32.47	30.78	27.67
BM3D	37.32	35.74	34.5	32.42	30.99
G3DDCT	34.82	34.10	33.28	31.29	29.71
GF	34.69	33.31	32.26	30.38	29.09
WNNM	37.90	36.34	35.14	32.96	31.39
IBM3DGF	38.74	37.62	36.68	34.84	33.43

**Figure 17:**
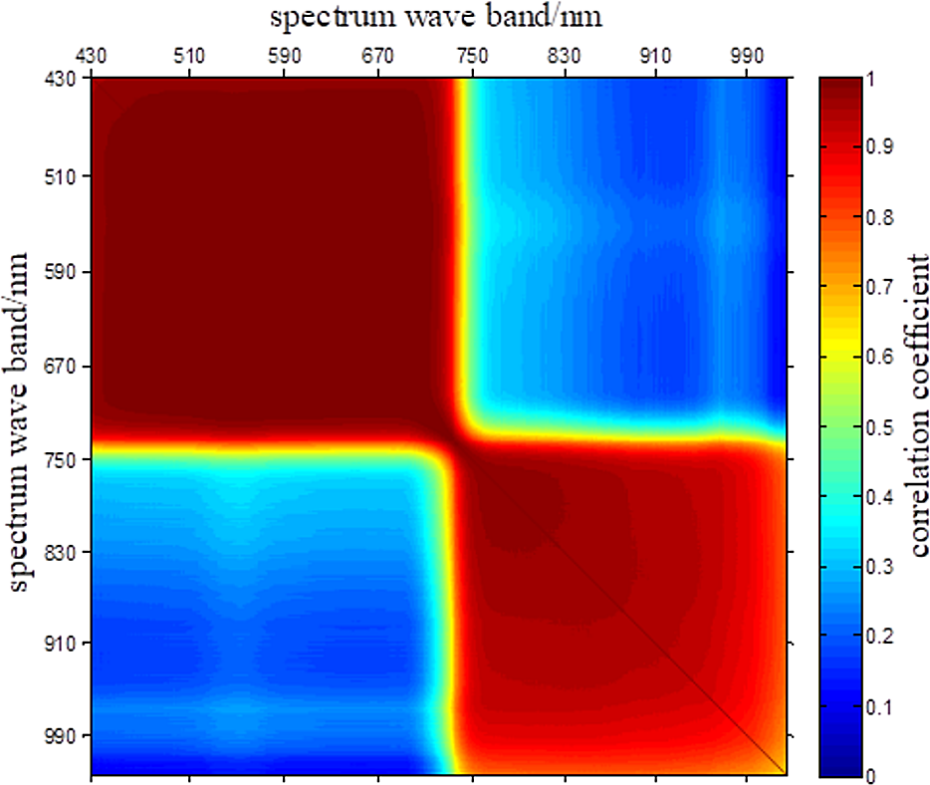
Spectral correlation coefficients of HSIs of camellia sinensis.

As shown in Fig. 17, a spectral correlation analysis was conducted on HSIs of camellia sinensis over the range of 430 nm–1,023 nm. As it can be seen that the correlation of the spectral curve of camellia sinensis in 430 nm–700 nm and 750 nm–1,023 nm is relatively high, and the correlation in 700 nm–750 nm is relatively low. Besides, the correlation among these three segments is also relatively low.

From the results of correlation analysis, 31.66% of slices are extremely correlated (—r—>0.95), 16.63% of slices are highly correlated (—r—>0.8), 5.78% of slices are moderately correlated (0.5 ≤—r—<0.8), and 45.92% of slices are barely (0.3 ≤—r—<0.5) or not correlated (—r—<0.3).

#### Noise estimation and analysis of local standard deviation

**Figure 18:**
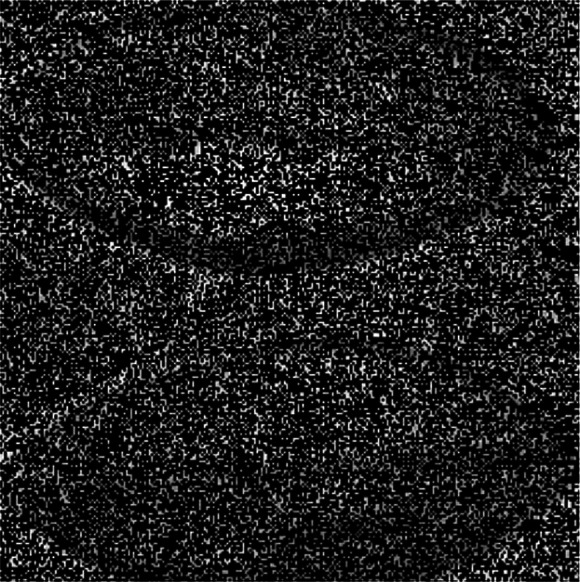
Noisy image.

**Figure 19:**
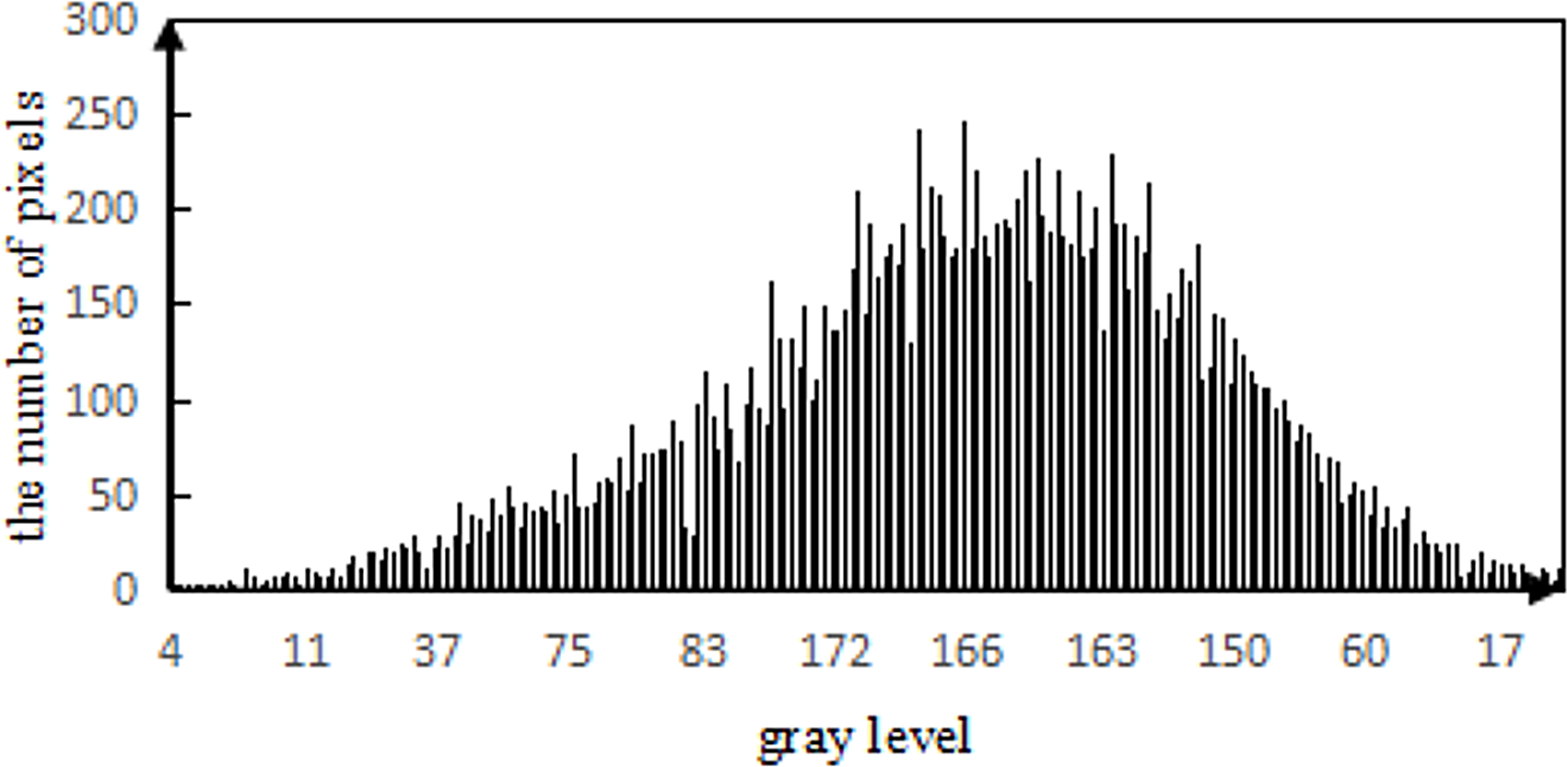
Gray histogram of noise.

According to the noise detection method of image evaluation without a reference, the 1,023 nm waveband of HSI of camellia sinensis is tested (Choi, Jung & Jeon, 2009). The noise figure is shown in Fig. 18 and the gray histogram of noise are shown in Fig. 19.

**Figure 20:**
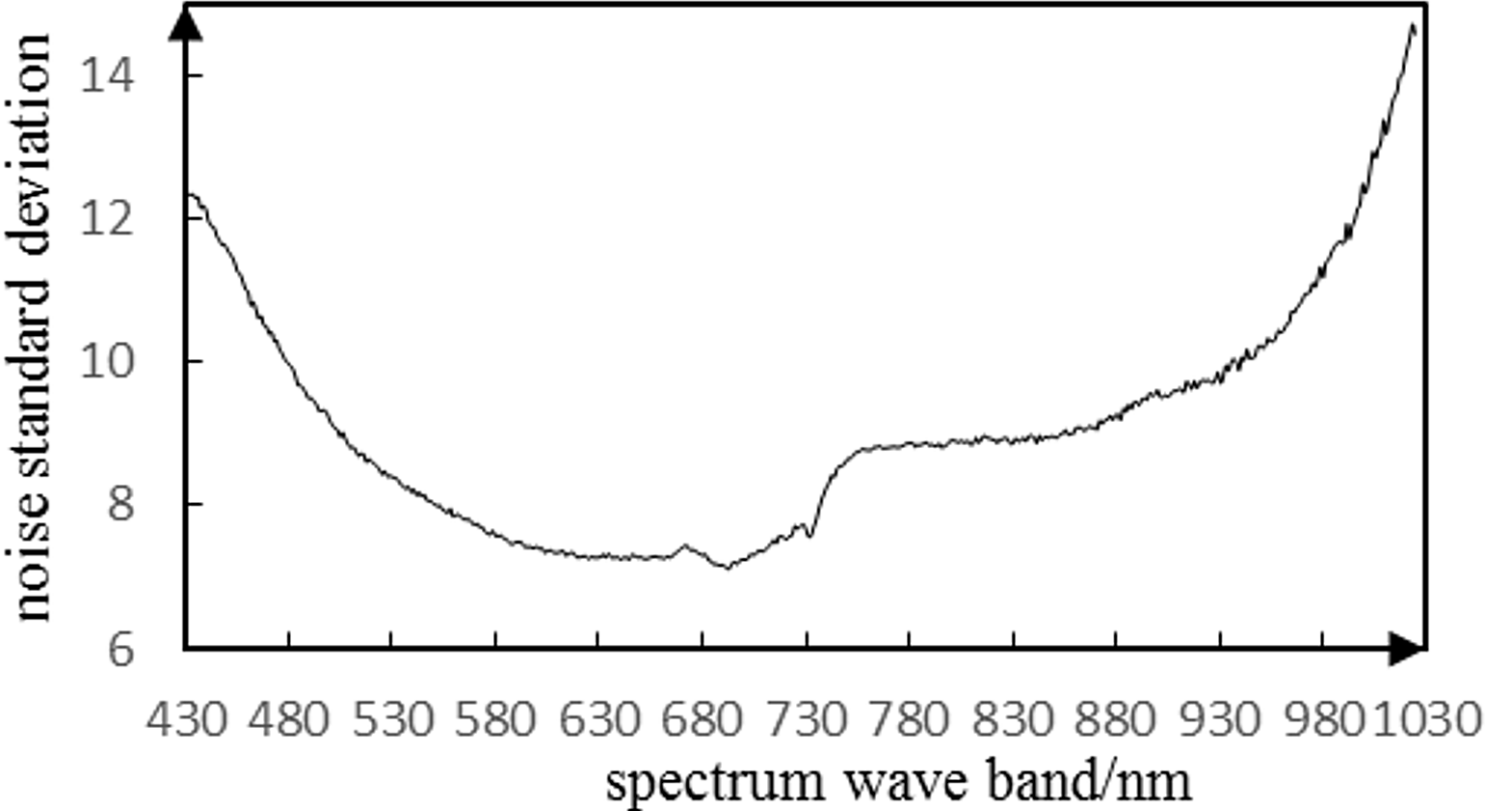
Estimation of hyperspectral noise of camellia sinensis based on the local mean standard deviation of eliminating edges.

The noise in HSIs of camellia sinensis is considered as a Gauss noise (Fig. 19). The noise standard deviation not only reflects the intensity of the noise, but also provides an important reference for the threshold selection. Given the noise intensity of HSI varies within the slice, a noise assessment method is needed to estimate the noise level of the HSIs (Liu, Bourennane & Fossati, 2012b). Gao (1993) proposed a method for assessing noise in HSI, which is called local mean and local standard deviation (LMLSD). Gao et al. (2007) put forward a local mean standard deviation method based on edge block culling to reduce the influence of image texture and edge on noise evaluation. The noise standard deviation of 469 spectral image slices over 430 nm–1,023 nm is estimated in Fig. 20.

 From the noise estimation curve of HSI of camellia sinensis, the slices at the range of 430 nm–530 nm and 930 nm–1,023 nm are affected by noise significantly. This henomenon appears consistent with the speculation in the analysis of spectral characteristics. The mean standard deviation is 10. 16 over the waveband range of 430 nm–530 nm, and 11.55 over the waveband rage of 930–1023 nm.

Assuming that one group of HSI of camellia sinensis has *w* slices, the data should be a $\sqrt{N}\times \sqrt{N}\times $ image cube. The prior knowledge of noise standard deviation is obtained by using the LMLSD method which is based on edge block culling. Also, the mean value of each standard deviation is used as the reference threshold in the denoising process.

### Evaluation of denoising performance in the spatia domain

**Figure 21:**
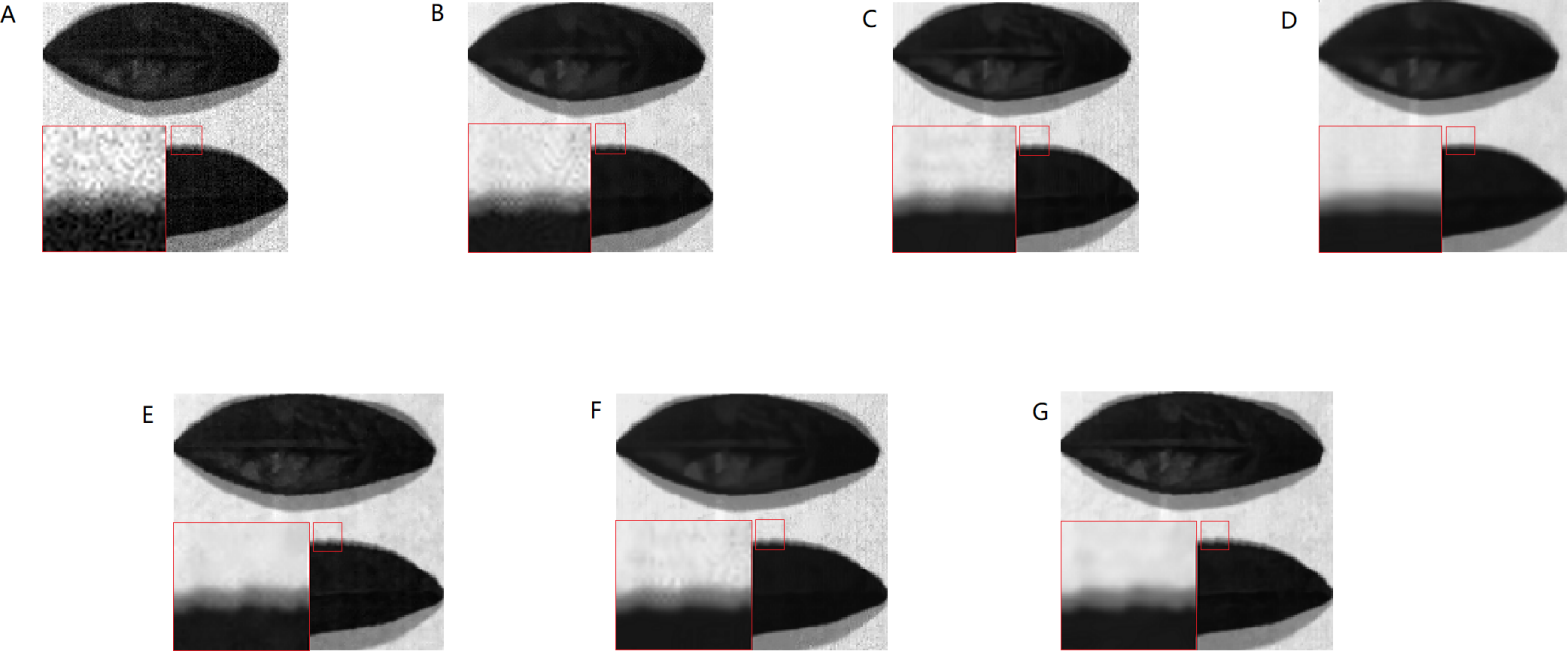
Comparison of different methods at the 435 nm band.

**Figure 22:**
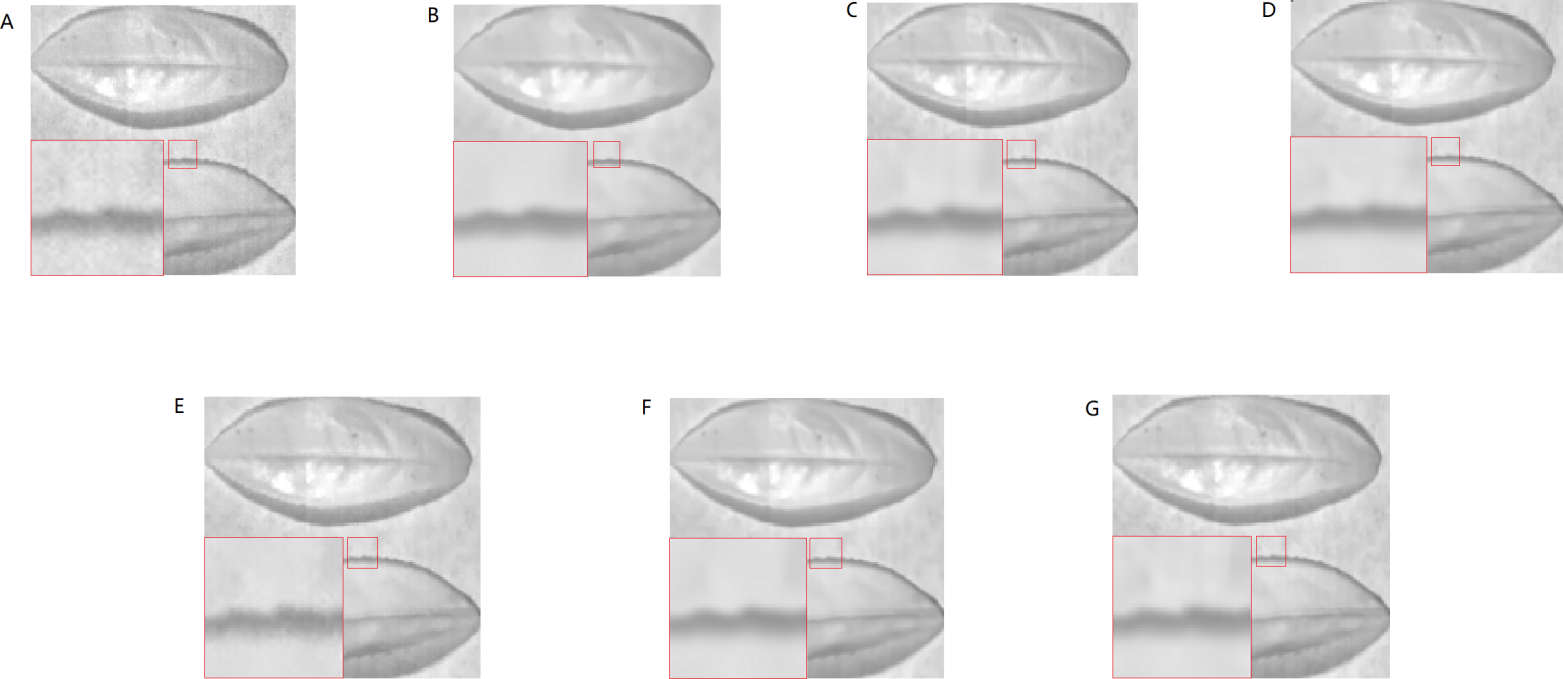
Comparison of different methods at the 840 nm band.

The real HSI of camellia sinensis included 430 nm–1,023 nm wavebands, in which 430 nm–530 nm and 930 nm–1,023 nm wavebands are severely affected by noise. The spectral image at 435 nm and 840 nm are chosen to make a comparison (Figs. 21A–21G–Figs. 22A–22G). BM3DGF not only makes full use of nonlocal correlation in the spatial domain and the characteristics of high inter-spectral correlation in the spectral domain, but also put intermediate band image which maintains detailed information about leaf veins and leaf area as the guide image to refine the HSIs. In the comparisons of the recovering partial enlargement for different denoising methods, IBM3DGF can also preserve the details and edge information of image better than the other denoising methods.

**Figure 23:**
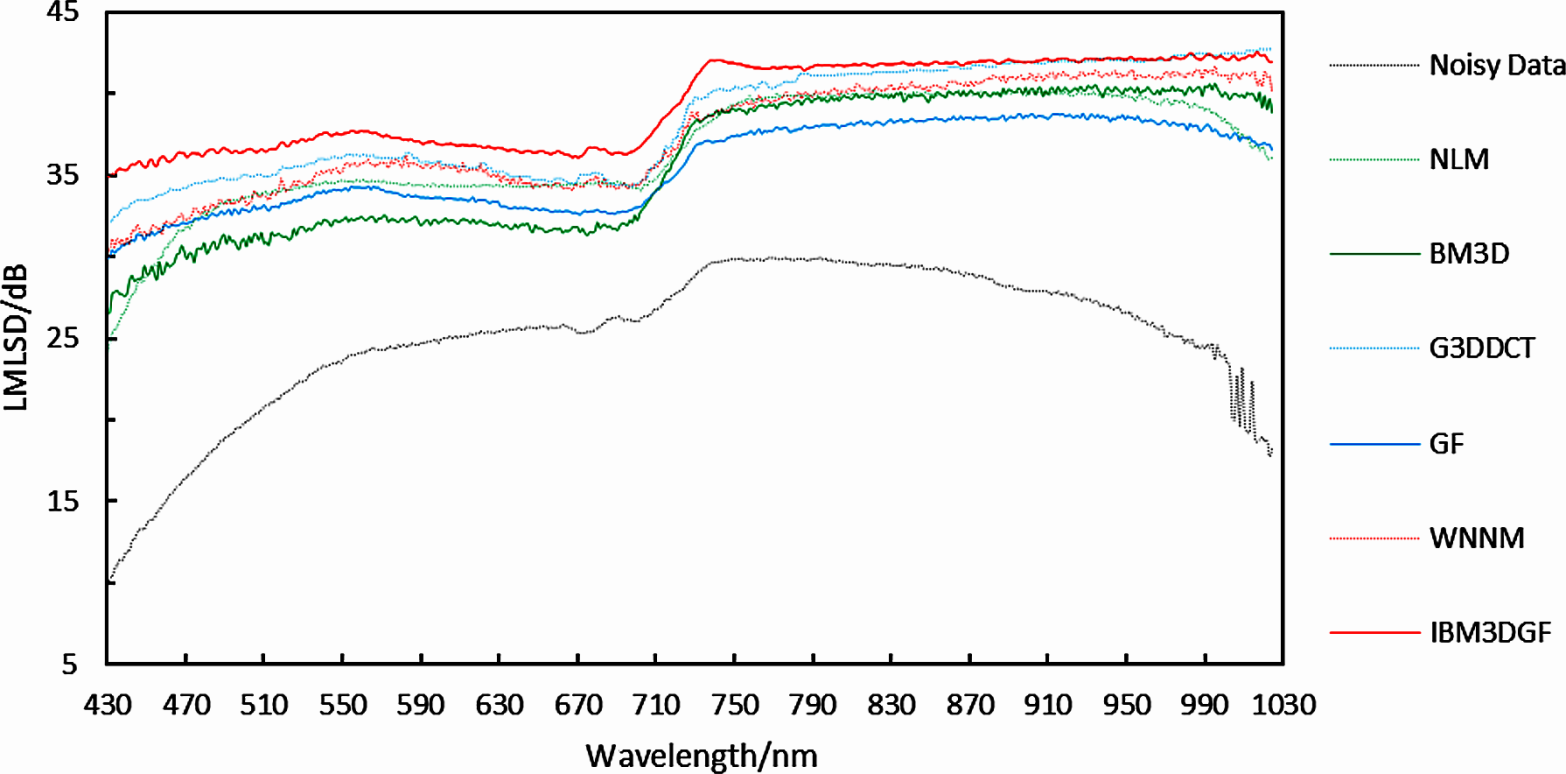
Curves of LMLSD of different denoising methods.

According to the denoising evaluation of the real HSIs, the detailed LMLSD-per-band performance of the proposed method is plotted in Fig. 23, along with the performance of the other methods. The proposed IBM3DGF method shows that the LMLSD-per-band is also higher than the other methods for most bands. The comparison of the mean LMLSD in the spatial domain for the real HSI is shown in Table 6. The mean LMLSD of the IBM3DGF is higher than that of the other methods. For the real HSI, the LMLSD of IBM3DGF is 1.01dB higher than that of G3DDCT. Therefore, the IBM3DGF method can achieve the best denoising results in the spatial domain for real HSIs, comparing with the other methods.

**Table 6:** Comparison of LMLSD in the spatial domain after denoising.

Different methods	LMLSD(dB)
Noisy data	25.03
NLM	36.51
BM3D	35.71
G3DDCT	38.35
GF	35.58
WNNM	37.44
IBM3DGF	39.36

### Evaluation of denoising performance in the spectral domain

**Figure 24:**
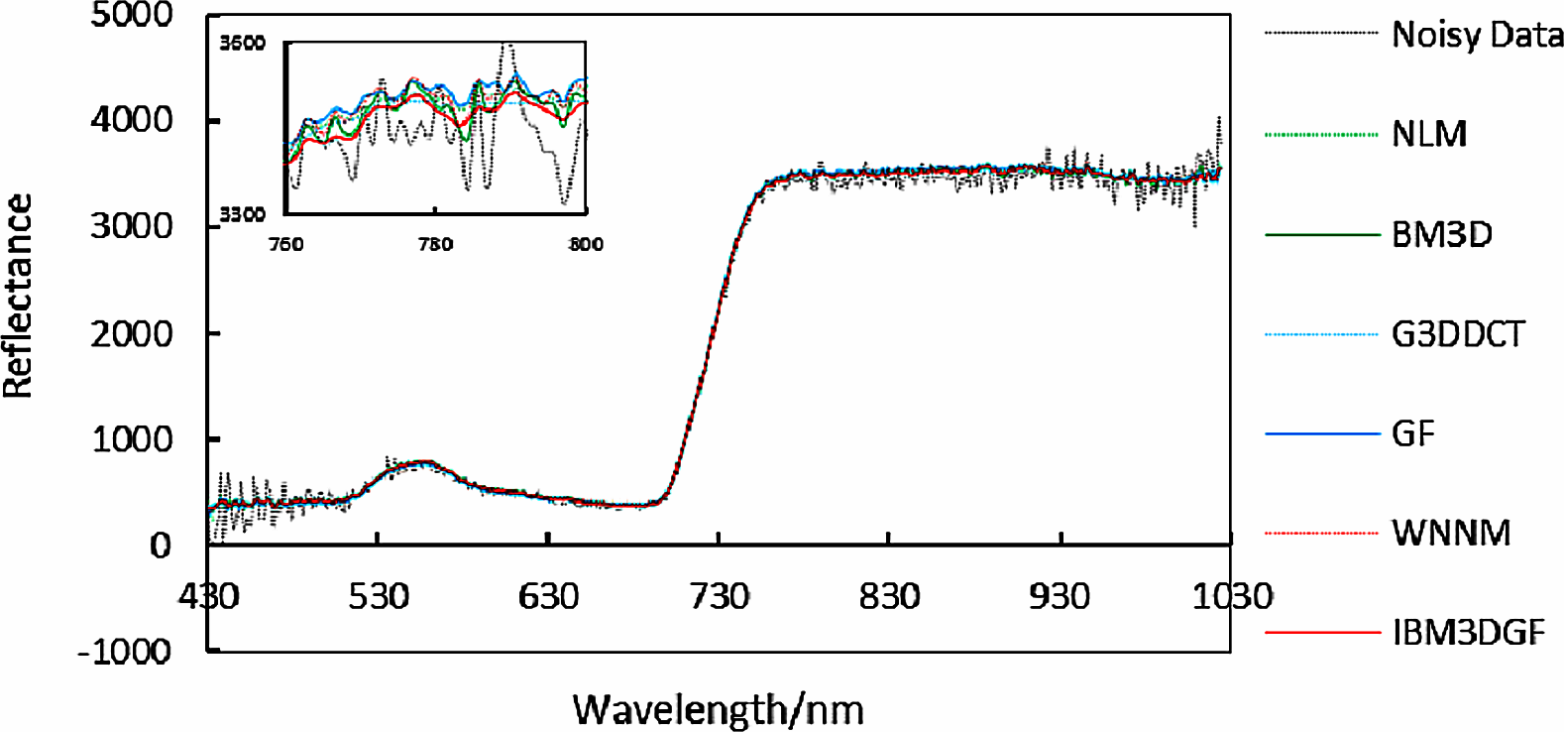
Comparison of spectral curves at *f* (85,170,*z*) for real data.

The experimental results of denoised spectral curves at *f*(85, 170, *z*) of the real HSIs by different methods are given in Fig. 24. Local images of spectral curves in the 430 nm–1,030 nm are magnified to be shown in the figures. It is shown that the spectral curves of IBM3DGF for real HSI data are all closer to the original curves than those of the other methods. IBM3DGF not only make full use of the high inter-spectral correlation and inter-spatial correlation, but also introduce a guided image filter to refine the details of the real HSIs.

**Figure 25:**
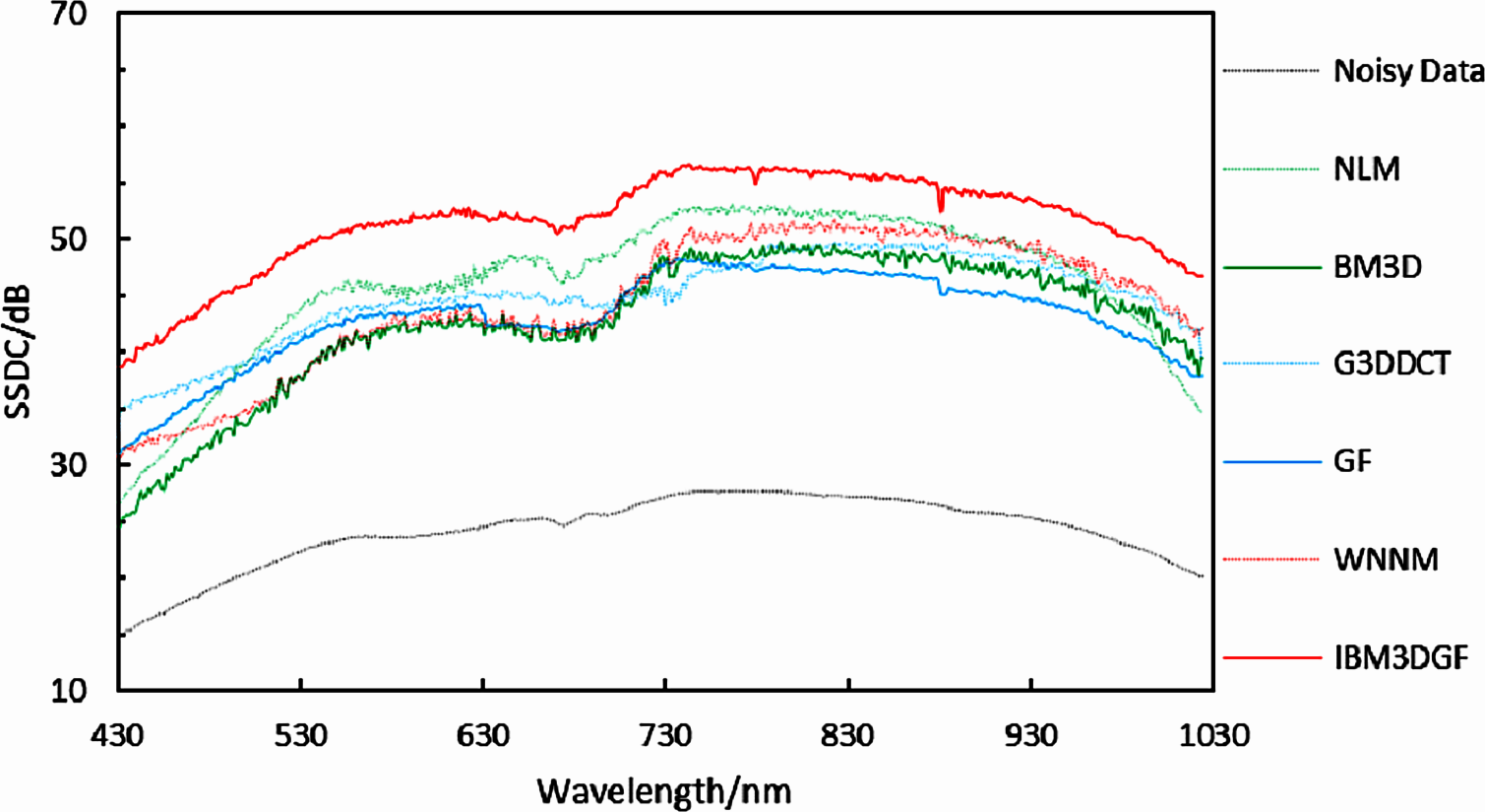
Curves of SSDC of different denoising methods.

Spatial spectral decorrelation (SSDC) (Gao et al., 2013b) is used to evaluate the noise of real HSI of camellia sinensis in the spectral domain. The core of SSDC is the signal removal of the high correlation between the spatial domain and spectral domain of HSIs by using multiple linear regression (Jiang & Wang, 2003).

The comparison of the SSDC in the spectral domain for the real HSIs is shown in Fig. 25 and Table 7. The SSDC of the IBM3DGF is significantly higher than that of the other methods. For real HSIs, the SSDC of IBM3DGF is 6.71dB higher than that of G3DDCT. Therefore, the IBM3DGF outperformed the other methods in the denoising effect.

**Table 7:** Comparison of SSDC in the spatial domain after denoising.

Different methods	SSDC/dB
Noisy data	24.13
NLM	46.04
BM3D	42.67
G3DDCT	44.79
GF	43.02
WNNM	44.32
IBM3DGF	51.50

## Discussion

In the IBM3DGF denoising result, not only the noise is thoroughly suppressed, but also the edge information and the detailed information are well preserved. In the result of NLM denoising method, some “artifacts” are produced with the image, and the edge information is also not well preserved. BM3D is one of the best denoising methods in designing for single image. G3DDCT divides the HSIs into groups and enhances the sparsity of HSIs. IBM3DGF induces the guided image filtering and combines the advantages of BM3D and G3DDCT denoising methods. IBM3DGF and WNNM can achieve better denoising performance than the other methods. From comparisons of the recovering partial enlargement for different denoising methods, IBM3DGF can preserve the details and edge information of image better than the other denoising methods. Therefore, comparison with the other denoising methods, the IBM3DGF method can not only maintain better edge information, but also suppress the noise information more effectively.

## Conclusions

In this study, a novel IBM3DGF denoising method for HSIs was proposed. inter-spectral correlation analysis is used to divided HSIs into several adjacent groups in which bands shows high coefficients. Adjacent three bands are interpolated to produce a high-resolution image which can utilize the inter-spectral correlation to a certain extent. BM3D is introduced to denoise the interpolated image. Guided image filtering method can enhance the denoising performance effectively for each group. Experimental results of the synthetic and real HSIs, the proposed IBM3DGF method can not only reduce spatial noises and preserve image details, but also suppress spectral noises effectively.

## Supplemental Information

10.7717/peerj.11642/supp-1 Supplemental Information 1 Source code

10.7717/peerj.11642/supp-2 Supplemental Information 2 Raw data exported from the Indian Pines Data Constant zero-mean additive Gauss white noise with the noise standard deviation of 15 for data analyses and preparation for Figs. 8, 9 and Table 1. Nonconstant zero-mean additive Gauss white noise with average noise standard deviation of 11.6 for data analyses and preparation for Figs. 9, 10 and Table 1.

## Additional Information and Declarations

### Data Availability

The following information was supplied regarding data availability: The codes and data are available in the Supplemental Files.
